# Types and Distribution of Bioactive Polyunsaturated Aldehydes in a Gradient from Mesotrophic to Oligotrophic Waters in the Alborán Sea (Western Mediterranean)

**DOI:** 10.3390/md18030159

**Published:** 2020-03-12

**Authors:** Ana Bartual, María Hernanz-Torrijos, Iria Sala, María J. Ortega, Cristina González-García, Marina Bolado-Penagos, Angel López-Urrutia, Leonardo Romero-Martínez, Luís M. Lubián, Miguel Bruno, Fidel Echevarría, Carlos M. García

**Affiliations:** 1 Facultad de Ciencias del Mar y Ambientales, Universidad de Cádiz, Puerto Real, 11510 Cádiz, Spain; mariahernanzt@gmail.com (M.H.-T.); iria.sala@uca.es (I.S.); mariajesus.ortega@uca.es (M.J.O.); marina.bolado@uca.es (M.B.-P.); miguel.bruno@uca.es (M.B.); fidel.echevarria@uca.es (F.E.); carlos.garcia@uca.es (C.M.G.); 2 Instituto Universitario de Investigaciones Marinas (INMAR), Campus de Excelencia Internacional del Mar (CEI-MAR), Universidad de Cádiz, Puerto Real, 11510 Cádiz, Spain; leonardo.romero@uca.es; 3 Departamento de Ecología y Gestión Costera, Instituto de Ciencias Marinas de Andalucía (ICMAN-CSIC), Puerto Real, 11510 Cádiz, Spain; cristinagg7@gmail.com; 4 Instituto Español de Oceanografía (IEO), Centro Oceanográfico de Gijón, 33212 Gijón, Asturias, Spain; lopezurrutia@gmail.com

**Keywords:** Alborán Sea, diatoms, oligotrophy, oxylipins, polyunsaturated aldehydes, PUA

## Abstract

Polyunsaturated aldehydes (PUAs) are bioactive molecules suggested as chemical defenses and infochemicals. In marine coastal habitats, diatoms reach high PUA production levels during bloom episodes. Two fractions of PUA can usually be analyzed: pPUA obtained via artificial breakage of collected phytoplankton cells and dissolved PUA already released to the environment (dPUA). In nature, resource supply arises as a main environmental controlling factor of PUA production. In this work, we monitored the vertical distribution and daily variation of pPUA associated with large-size phytoplankton and dPUA, at three sites located in the Alborán Sea from mesotrophic to oligotrophic waters. The results corroborate the presence of large-size PUA producers in oligotrophic and mesotrophic waters with a significant (58%–85%) diatom biomass. In addition to diatoms, significant correlations between pPUA production and dinoflagellate and silicoflagellate abundance were observed. 2*E*,4*E*/*Z*-Heptadienal was the most abundant aldehyde at the three sites with higher values (17.1 fg·cell^−1^) at the most oligotrophic site. 2*E*,4*E*/Z-Decadienal was the least abundant aldehyde, decreasing toward the oligotrophic site. For the first time, we describe the daily fluctuation of pPUA attributable to cellular physiological state and not exclusively to taxonomical composition. Our results demonstrate the persistence of threshold levels of dPUA deep in the water column, as well as the different chromatographic profiles of dPUA compared with pPUA. We propose different isomerization processes that alter the chemical structure of the released PUAs with unknown effects on their stability, biological function, and potential bioactivity.

## 1. Introduction

Oxylipins are organic biomolecules released by terrestrial and aquatic photosynthetic organisms [1]. These molecules are produced from lipoxidation of polyunsaturated fatty acids (PUFAs), and many of them are volatile compounds that contribute to the “smell” and “taste” characteristics of producers. In flowering plants, which lack C20:4 fatty acids, oxylipins are produced mainly from the C18 linolenic (18:3) and linoleic (18:2) fatty acids [2,3,4]. Aldehydes, such as hexanal or 2E,4*E*-decadienal, are commonly produced odorants. In terrestrial environments, these flavor compounds are rapidly volatilized in the air, interacting with terrestrial organisms via olfaction uptake. In aquatic environments, phytoplankton also produce oxylipins, particularly aldehydes derived from C20 fatty acids [5,6]. In these environments, solubility, instead of volatility, is crucial for the persistence of these compounds in the waters surrounding producers, interacting biologically with neighboring organisms via direct contact, instead of olfaction. Among phytoplankton, diatoms are the most widely distributed group in natural aquatic environments and constitute the main primary producers in coastal marine habitats [7]. This photosynthetic group produces distinctive volatile long-chain oxylipins, called polyunsaturated aldehydes (PUAs) [6,8,9], after cell wounding. These PUAs (e.g., 2*E*,4*E/Z*-heptadienal, *2E,*4*E/Z*-octadienal, or 2*E*,4*E*,7*E/Z*-octatrienal) are mainly derived from lipoxidation of essential fatty acids as eicosapentanoic acid (EPA) and docosahexanoic acid (DHA) [10]. Although PUAs are not the most abundant oxylipins produced, they were experimentally demonstrated to have a teratogenic effect on copepods [11,12], as well as stimulation predation on ciliates by copepods [13]. In nature, differential effects of PUAs on the bacterial community [14,15,16] and ciliates [17] were reported, although their role and ecological significance remain poorly understood. Reference [18] demonstrated that polyunsaturated aldehydes produced after diatom cell wounding act as a contact poison by accumulating in organism cell membranes due to their hydrophobicity. These molecules, once released by the diatom cells, become part of the available dissolved organic carbon pool (DOC) for bacterioplankton remineralization [16]. Physicochemical characteristics of the water mass (i.e., salinity, temperature, turbidity, pH) should also regulate their spatial distribution, persistence, chemical characteristics, and/or area of action (classically “distance sense”) in aquatic environments. However, it remains to be analyzed whether these compounds would persist long enough to have any potential effect on non-producer phytoplankton, bacterioplankton, or zooplankton as experimentally tested. Notwithstanding, during the last two decades, substantial research went into determining their biological functions in nature [13,14,17,19,20,21].

PUAs show effects on bacterial growth and metabolic activity [14], as well as zooplanktonic organisms [13,22,23,24], in μM concentrations. These ranges were only experimentally quantified as the particulate fraction (pPUA) after artificial cell breakage for PUA quantification from algal dense cultures or samples collected during a diatom bloom in the Adriatic Sea [25,26]. Nevertheless, they were never measured for the dissolved fraction in natural seawater. In contrast, μM ranges of dissolved PUA (dPUA) are expected to occur in the immediate vicinity of the producer cell but disperse rapidly. This is consistent with pM to nM concentrations of dPUA quantified in marine environments, such as the Strait of Gibraltar [27,28]. Persistence of dPUAs in natural waters is an important feature once they are produced after cell breakage. The pre-exposition to low levels (pM or nM) as a dissolved fraction in the water column could confer (or not) resistance to micromolar levels. This could also imply a competitive advantage of pre-exposed species against non-previously exposed species during events of high bloom production as previously observed [29]. Nanomolar concentrations of PUA were shown to have an effect on microzooplankton grazing preferences [17]. In this case, the pelagic community structure is liable to be affected by top-down regulation, by reducing grazing pressure and altering trophic networks, especially after diatom-PUA producer bloom events. 

Collecting field data to discern PUA’s ecological distribution and significance was one aim during the last decade. A macroecological pattern of pPUA distribution was found, showing the higher production of pPUA especially by small-size (<10 μm) phytoplanktonic cells collected in oligotrophic waters (total chlorophyll <1 mg·m^−3^) [30]. Regarding dPUA, relatively high concentrations (nM) of these compounds were also shown to define patches in the surface layer of coastal waters under non-blooming and mixing conditions [28]. In this work, we analyze pPUA and dPUA at three natural Mediterranean sites subjected to different resources conditions. The Mediterranean Sea is usually subjected to oligotrophic conditions [31] with nutrient deficit as a consequence of a net nutrient loss in the budget between the Mediterranean Sea and the Atlantic Ocean through the Strait of Gibraltar [32]. This nutrient limitation is usually compensated by river runoff, atmospheric depositions, and diazotroph fixation [33,34], as well as an important vertical circulation and winter mixing in some locations. Physical processes, such as internal tides and the displacement of the Atlantic–Mediterranean interface can also inject nitrate and phosphate into the upper layer [32,35]. As a consequence, there is a complex spatio-temporal distribution of trophic regimes in this sea, with strong oligotrophic areas together with highly eutrophized areas, such as the Northern Adriatic Sea. We sampled at three sites located in the Alborán Sea, a western Mediterranean area that receives waters from the Atlantic Ocean through the Strait of Gibraltar, where ranges of pPUA were previously assessed [27]. We focused our sampling effort in three highly dynamic stations where a gradient of eutrophic-to-oligotrophic state from coast to offshore was expected. The aim was to find correlations of pPUA with phytoplankton community composition and its physiological state. A high-resolution survey was performed, paying special attention to the vertical distribution and daily patterns of the dissolved fraction of these bioactive compounds (dPUA) in the selected natural sites, looking specifically at correlations with physicochemical characteristics of the water column. This will provide novel information regarding the persistence and biological origin of PUAs for a better understanding of their ecological role.

## 2. Results

### 2.1. Trophic Status of the Different Sites and Physicochemical Characterization of the Water Mass 

**Figure 1:**
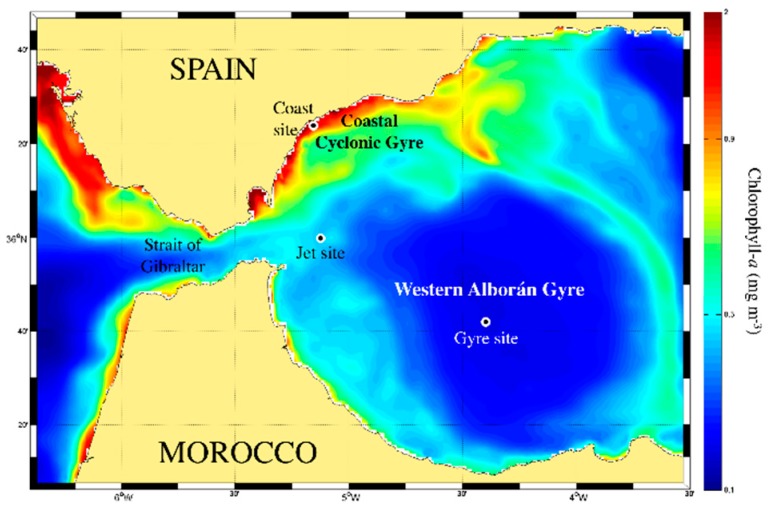
Map showing the location of the different sampling sites named Coast, Jet, and Gyre sites, with overlaid mean chlorophyll a concentration (mg·m^−3^) for the sampling period (from 7–9 October 2015). Chlorophyll a data were downloaded from the Copernicus Marine Environmental Monitoring Service (CMEMS; http://marine.copernicus.eu/).

Ocean color images in the Alborán Sea were downloaded from 7–9 October 2015, showing the distribution of mean sea surface chlorophyll *a* concentration (Chl*a*; mg·m^−3^) during the cruise (Figure 1). These images were based on merged satellite data (SeaWIFs, MODIS-Aqua, MERIS, VIIRSN, and OLCI-S3A) using optimal interpolation to get images of daily Chl*a* concentration (mg·m^−3^), at a full spatial resolution of ∼1 km. As shown in Figure 1, this image evidenced the chlorophyll gradient that characterizes the three sampling sites examined in this work: Coast, Jet, and Gyre sites. 

Additionally, for a better characterization of the trophic status of the three stations, we used the trophic index Fp defined by Reference [35] on the basis of the pigment concentrations obtained via HPLC analysis from discrete samples collected at different depths. We found an averaged value of Fp index of 0.770 ± 0.070 for the Coast site (Table 1), which is typical for Mediterranean mesotrophic conditions, compared with a much lower averaged index of 0.073 ± 0.095 for the Jet site, and 0.130 ± 0.138 for the Gyre site, which are in the range of North Atlantic and Mediterranean oligotrophic regimes, respectively. 

**Figure 2:**
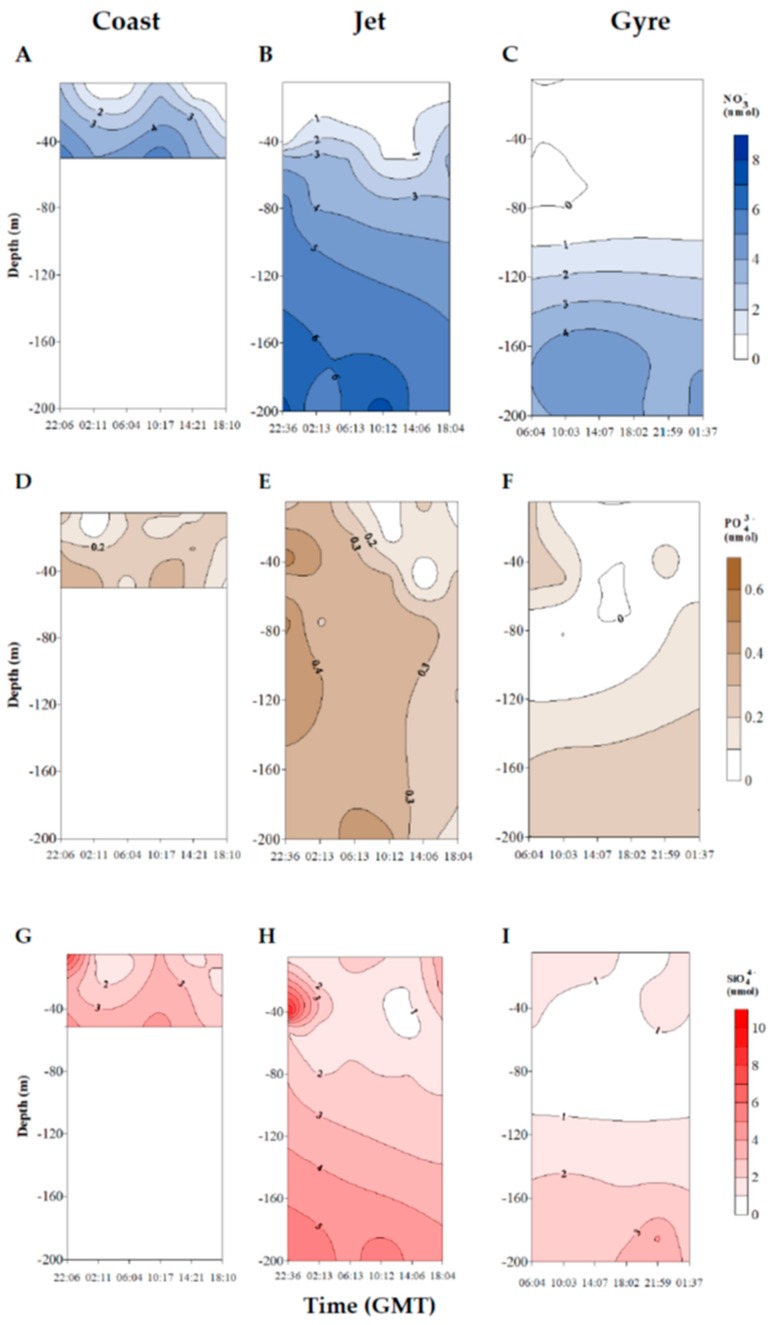
Vertical distribution of nutrients concentrations along the sampling day (μM) (NO_3_^−^, (**A**–**C**); PO_4_^3−^, (**D**–**F**); SiO_4_, (**G**–**I**)) from surface to 200 m at the Coast, Jet, and Gyre sites.

Nutrient concentrations increased with depth at the three sites (Figure 2). The Gyre site showed the lowest average concentrations of all nutrients, especially PO_4_^3−^ (Table 1). In fact, the PO_4_^3−^ concentration was zero in several samples. The averaged molar ratio NO_x_:PO_4_ obtained for the Gyre site was 25, far from the N:P (16:1) Redfield ratio (Table 1). This is indicative of a trend for the limitation status of this station during our sampling period. The Jet and Coast sites did not show significant differences in NOx (NO_3_^−^ + NO_2_^−^), PO_4_^3−^, and SiO_4_ concentrations, and both were significantly higher compared with the Gyre site (Kruskal–Wallis test; *p* < 0.001) (Table 1), with important fluctuations along the sampling day (Figure 2). Meanwhile, the averaged stoichiometric SiO_4_:NOx molar ratio was over 2 at the Jet and Gyre stations, such that the supply of silicate relative to nitrate seemed to be sufficient (Si:N ratio = 1:1) (Table 1). In the coastal station, the SiO_4:_NOx ratio was slightly lower (1.76 ± 1.57). These results corroborated a mesotrophic-to-oligotrophic status gradient from the Coast to the Gyre, with phosphorus limitation in the Gyre. 

Three water masses were vertically identified at the different sites: the Surface Atlantic Water (SAW) with temperature between 19–20 °C and salinity of ~36, the North Atlantic Central Water (NACW) with temperature ~13.6 °C and salinity ~35.8, and the colder and saltier deeper Mediterranean Water (MW) with a temperature of ~13 °C and salinity of ~38 (Table 1). A salinity gradient was observed at the Jet and Gyre sites at 120 and 200 m depth, respectively (Table 1).

Regarding turbulence conditions, the rate of dissipation of turbulent kinetic energy (ε, m^2^·s^−3^) decreased with depth at the three sites, with maximal values at surface at the Jet site (× 10^−5^) and minimal at depth >50 m at the Gyre site (× 10^−9^). Total averaged ε was lower at the Gyre site compared with the other sites (Table 2), although statistically significant differences were not established between sites (Kruskal–Wallis test: *p* = 0.0194). However, when data were compared for a maximal depth of 50 m, ε was significantly affected by site, depth, and their interaction (Table 2), being lower at deeper waters compared with surface and at the Gyre site compared with the other two sites.

### 2.2. Polyunsaturated Aldehydes 

#### 2.2.1. Particulate PUA (pPUA > 10 μm): Type and Distribution

**Figure 3:**
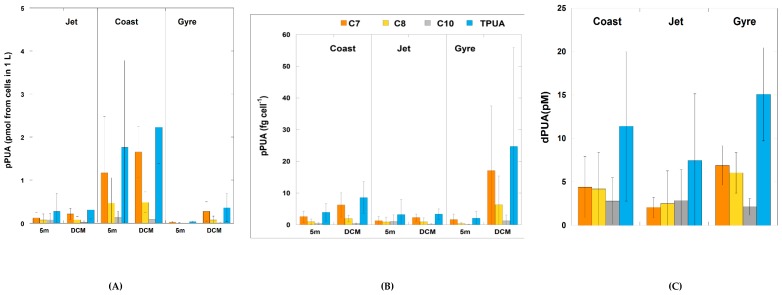
Different fractions of polyunsaturated aldehydes (PUAs) at the three sites: (**A**) averaged particulate PUA (pPUA of large-size phytoplankton) expressed as pmol from cells in 1 L at 5 m and deep chlorophyll maximum (DCM). (**B**) pPUA normalized by large-size phytoplankton cell abundance (fg PUA·cell^−1^) at 5 m and DCM; (**C**) averaged vertical dPUA (pM) at the three sites. C7: 2*E*,4*E*/*Z*-heptadienal; C8: 2*E*,4*E*/*Z*-octadienal; C10: 2*E*,4*E*/*Z*-decadienal; TPUA: total PUA.

PUA producers at surface and deep chlorophyll maximum (DCM) were detected at all sampled sites, with an average total pPUA concentration ranging from 0.02 to 5.3 pmol from cells in 1 L (Figure 3A). Maximal values were obtained at the DCM of the Coast site (5.30 pmol from cells in 1 L) and the lowest at surface of the Gyre site (0.02 pmol from cells in 1 L) with significantly higher concentrations at the Coast site (one-way ANOVA; *p* < 0.0001) throughout the entire sampling period, mainly at DCM depth (Figure 3A). 

Three main different aldehydes were identified and quantified: 2*E*,4*E*/*Z*-heptadienal (pC7), 2*E*,4*E*/*Z*-octadienal (pC8), and 2*E*,4*E*/*Z*-decadienal (pC10), although traces of others were also observed as 2*E*,4*E*,7*E*/*Z*-octatrienal or 2*E*,4*E*,7*E*/*Z*-decatrienal, but their abundance was very low. pC7 was the most abundant aldehyde at the three sites (Figure 3A), especially at the DCM, with significantly higher concentrations at the Coast site (one-way ANOVA; *p* < 0.0001) in comparison with the other two sites at both depths. pC8 was also significantly more abundant at the Coast site compared with Jet or Gyre (one-way ANOVA; *p* < 0.01). Meanwhile, pC10 was the least abundant aldehyde with slightly higher concentrations at the Coast and Jet sites (one-way ANOVA; *p* < 0.05) compared with the Gyre site (Figure 3A). 

Because pPUAs are obtained from artificial breaking of large size phytoplanktonic collected cells (>10 μm), these data were normalized to large-size phytoplankton cell abundance to obtain pPUA·cell^−1^ assuming that all collected cells are PUA producers. No significant differences could be established among sites (one-way ANOVA; *p* = 0.297). By aldehyde, at 5 m, no significant differences were obtained between sites (Figure 3B), however, the Gyre DCM stood out with significantly higher values of pC7 (fg·cell^−1^) compared with the two other sites (Figure 3B) (one-way ANOVA; *p* < 0.05). 

Correlations among pPUA concentrations of the whole dataset showed that pC8 was significantly and positively correlated with pC7 and pC10 (Table 3), while pC7 and pC10 were not correlated (Table 3). By site distinction, there were found different trends. pC7 and pC8 were significantly and positively correlated at the Coast site (Spearman rank correlation; *r* = 0.98; *p* < 0.05) and Gyre site (Spearman rank correlation; *r* = 0.84; *p* < 0.05); however, this correlation was missed at the Jet site. pC10 was significantly and positively correlated with pC8 at the Jet site (Spearman rank correlation; *r* = 0.68; *p* < 0.05) and Gyre site (Spearman rank correlation; *r* = 0.66; *p* < 0.05), while they were not correlated at the Coast site. pC7 and pC10 were not correlated at any site. 

#### 2.2.2. Dissolved PUA (dPUA): Types and Distribution

Three different aldehydes were identified and quantified as for pPUA: 2*E*,4*E*/*Z*-heptadienal (dC7), 2*E*,4*E*/*Z*-octadienal (dC8), and 2*E*,4*E*/*Z*-decadienal (dC10). Total dPUA ranged from a minimal concentration of 1.33 pM at the Gyre site to a maximal of 34.86 pM at the Coast site. Averaged total dPUA was 11.41 ± 8.59 pM at the Coast site, 7.46 ± 7.67 pM at the Jet site, and 15.08 ± 6.367 pM at the Gyre site (Figure 3C). No statistically significant differences were observed for averaged total dPUA between sites (one-way ANOVA; *p* = 0.071). However, based on type of aldehyde, dC7 was significantly lower at the Jet site (multiple comparison p-value (two-tailed); *n* = 84; *p* < 0.0001) compared with the other two sites, while no significant differences were observed between the Coast and Gyre site for averaged dC8 and dC10. By percentage of abundance, the relative abundance (%) of dC10 was significantly lower at the Gyre (*t*-test; *p* < 0.00001) with respect to the other two sites. The averaged concentrations of the three aldehydes were not significantly different at the Jet site (*t*-test; *p* > 0.05) (Figure 3). At the Gyre site, the dC10 concentration was significantly lower compared with dC8 and dC7 (*t*-test; *p* < 0.000001).

**Figure 4:**
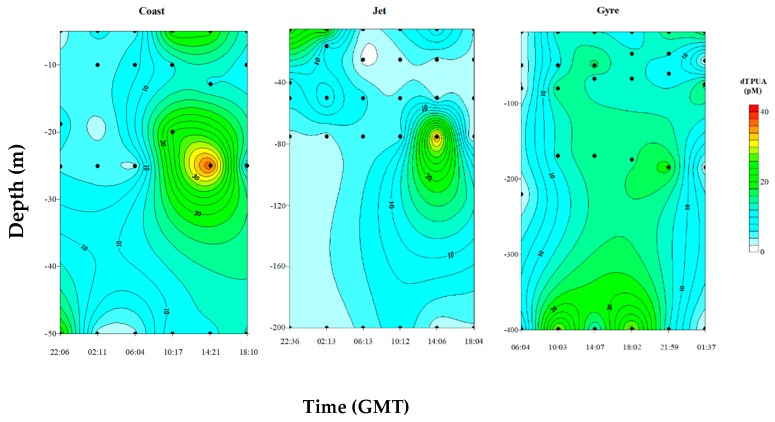
Daily vertical distribution of total dissolved PUA (dTPUA) at the three sites. Please note that depth scales are different for each site for a better observation of dPUA patterns. The black dots indicate sampling depths.

Figure 4 shows the temporal evolution of vertical distribution of dPUA for the three sites. At the Coast and Jet sites, the dPUA distribution defined clear patches with distinguished maxima. These maxima were observed deeper than the DCM that was located at 13.66 ± 4.73 m at the Coast site and 27.33 ± 11.68 m at the Jet site. These maxima did not persist much in time throughout the day (Figure 4). The vertical distribution of dPUA at the Gyre site was different than that observed at the two other sites, showing a wider distribution of averaged higher concentrations, without defining clear patches, and with a longer daily persistence. 

We found positive and significant correlations between dC7 and dC8 for the whole dataset without site distinction (Spearman rank correlation *r* = 0.800; *p* < 0.01) (Table 3). By site, dC7 and dC8 were positive and significantly correlated at the Coast (*r* = 0.85; *p* < 0.01) and Jet sites (*r* = 0.92; *p* < 0.01) but not at the Gyre site. dC10 was only correlated with dC7 and dC8 at the Jet site with *r* > 0.9 (*p* < 0.01). At the Gyre site, no significant correlations were established between aldehydes.

Two types of PUA isomers for both pPUA and dPUA fractions were distinguished. One type involved geometrical isomers at the C-2 and C-4 positions of the aliphatic chain of PUA (Type I), which included *E/E*, *E/Z*, *Z/E*, and *Z/Z* isomers (Figure S1, Supplementary Materials), whose production was attributed to diatoms [36]. A second type of isomer (Type II) became abundant in several natural samples. As shown in Table 4, the percentage of abundance of both types of isomers (Type I and II) differed among the particulate and dissolved fractions of PUA. The percentage of Type II isomers was significantly higher (one-way ANOVA; *p* < 0.001) for dPUA than for pPUA at all sites and both depths (5 m and DCM), and this was the general trend for the three aldehydes (C7, C8, and C10). For the pPUA fraction, the three aldehydes showed different trends. Most pC7 isomers were Type I, while they were Type II for pC8 and more balanced for pC10, with approximately 50/50 abundance (Table 4). 

### 2.3. Chlorophyll Fluorescence and Pigments Concentrations

**Figure 5:**
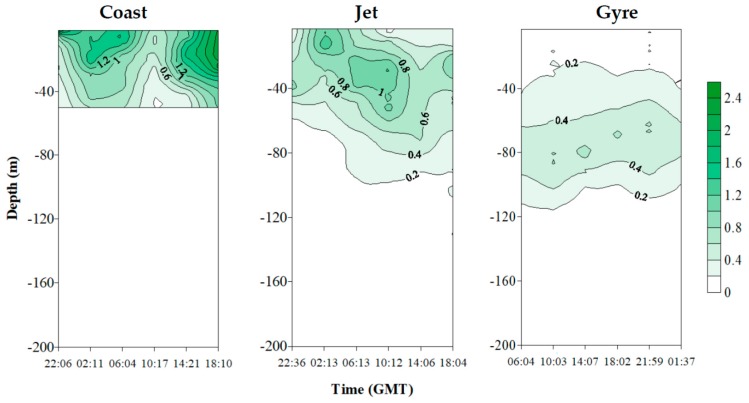
Vertical distribution scaled from 0 to 200 m of in situ fluorescence at the Coast, Jet, and Gyre sites.

Maximum values of fluorescence (mg·m^−3^) were recorded at the Coast site, decreasing toward the center of the Gyre (Figure 5). This coast–offshore gradient agrees with the superficial distribution of chlorophyll obtained from satellite data (Figure 1). Additionally, the vertical profiles of fluorescence showed how the depth of maximum chlorophyll (deep chlorophyll maximum or DCM) deepened with this coast-to-offshore gradient, from mesotrophic to oligotrophic. The maximum of chlorophyll at the Coast site was found at 15–25 m, at the Jet site at 40–50 m, and at the Gyre site at 70–75 m (Figure 5). 

Concentrations of total Chl*a* (TChl*a*) from discrete samples for all sites ranged from 0.01 to 0.54 mg·m^−3^ for the whole sampling period, which is indicative of non-blooming conditions during the sampling dates (Table 5). Maximum values were observed at the DCM at the Jet site (0.67 mg·m^−3^), and at surface waters at the Coast site (0.54 mg·m^−3^). The Coast site showed an averaged TChl*a* of 0.27 ± 0.15 mg·m^−3^ for the whole sampling period and both depths. Those values were very similar to the averaged TChl*a* concentration observed at the Jet site (0.26 ± 0.20 mg·m^−3^), with lower TChl*a* concentrations at the surface compared with the DCM (Table 5). The lowest TChl*a* concentrations were obtained at the Gyre site with an averaged concentration of 0.07 ± 0.06 mg·m^−3^ (Table 5).

HPLC pigments can be used as taxonomic biomarkers for the different phytoplankton size classes [37,38,39], and their ratios can give information regarding their relative abundance. Several ratios were calculated between different HPLC-determined pigments (Table 5). These results showed an important diatom pigment abundance (ratio DT/TChl*a* > 1) at the Coast site (1.77 ± 0.49) throughout the whole sampling period, at surface and DCM depths (Table 5). At the Jet site, the averaged DT/TChl*a* ratio was 0.91 ± 0.248, also indicative of a high percentage of abundance of diatoms at both sampled depths. On the basis of Chl*c* concentrations, other chromophytes (Chl *c-*containing algae) other than diatoms should be present at the Jet and Coast sites. Contrary to this trend, at the Gyre site, the ratio DT/Chl*a* was significantly higher at the surface than at the DCM (Table 5), with important fluctuations along the sampling day. This denotes a lower diatom abundance at the DCM at the Gyre. In fact, at the DCM in the Gyre site, the 19’-hex/Chl*a* ratio ranged from 0.3 to 6.7 (average 1.88 ± 2.071) suggesting the importance of other nanoflagellates than diatoms for the micro and nanoplanktonic fraction [40]. 

### 2.4. Phytoplankton Composition

#### 2.4.1. Small-Size (<10 μm) Phytoplankton Abundance and Biovolume

Four categories of small-size phytoplankton (< 10 μm) were distinguished on the basis of flow cytometry analysis: *Phrochlorococcus*, *Synechococcus*, Nanoeukaryotes, and Picoeukaryotes. No significant differences were observed in *Prochlorococcus* averaged cell abundance (one-way ANOVA; *p* = 0.9786), nor in *Synechococcus* (one-way ANOVA; *p* = 0.6477) among the three sites (Table 4). However, there was a vertical gradient in cyanobacterial cell abundance (i.e., *Prochlorococcus* + *Synechococcus*) at each site, with maximum values at the DCM (Table 6). By percentage of abundance, the small-size phytoplankton fraction (< 10 μm) was dominated by cyanobacteria (i.e., *Prochlorococcus* + *Synechococcus*) at the Gyre site (91.64%) and was also quite abundant at the Jet site (74.48%). However, the minimum contribution of cyanobacteria was found at the Coast site (52.81%). Meanwhile, at the Coast site, the percentages of abundance of Picoeukaryotes (40.39%) and Nanoeukaryotes (6.80%) were significantly higher (*t*-test; *p* < 0.00001) compared with the other two sites.

#### 2.4.2. Large-Size (10–250 μm) Phytoplankton Abundance and Biovolume

On the basis of FlowCAM analysis, we found that collected samples at the Coast and Jet sites showed an averaged cell abundance of diatoms of 61.50% ± 0.87% and 46.40% ± 1.93% of total large-size phytoplankton, respectively, with dinoflagellates and coccolithophorids being less than 4% of total large phytoplankton cell abundance throughout the sampling period at both depths. Moreover, an important fraction of non-identified phytoplankton of the smaller size range was observed (>30 %) throughout the sampling period at the Coast and Jet sites at both depths (Table 7). At the Coast site, typical diatom PUA producers such as the genus *Skeletonema* were abundant among other considered diatom categories, with cell abundances that ranged from 2.9 × 10^5^ to 1.9 × 10^7^ cell·L^−1^ especially at the surface. Together with this genus, other diatom genera such as *Thalassionema* and *Chaetoceros* were also abundant. At the Jet and Gyre sites, a slight increase in dinoflagellate abundance was observed compared with the Coast site (Table 7). The genus *Skeletonema* (1.39 × 10^6^ ± 1.1 × 10^6^ cell·L^−1^) and *Thalasionema* (1.75 × 10^6^ ± 1.38 × 10^6^ cell·L^−1^) were the most abundant diatoms at surface and DCM depths at the Jet site, together with the diatom specie *Guinardia striata* (8.4 × 10^5^ ± 7.98 × 10^5^ cell·L^−1^). These results are consistent with the observed trends obtained from HPLC pigment analyses explained above.

Large-size phytoplankton cell abundance differed significantly at the Gyre site, where diatom percentage decreased considerably to a total average of 17.38% ± 5.94% throughout the sampling period (Table 7), and especially at the DCM with values under 8% in several samples. Compared with the other two sites, large phytoplankton at the Gyre had a higher percentage of relative cell abundance of dinoflagellates (15.09% ± 4.48%) and coccolithophorids (3.69% ± 2.12%) (Table 7). Diatoms never reached cell densities >10^5^ cell·L^−1^ and many categorized diatom classes were totally absent in several samples. *Skeletonema* was present with cell densities that ranged from 6.96 × 10^4^ to 7.98 × 10^4^ cell·L^−1^ at both sampled depths. The highest cell abundance (6.1 × 10^6^ cell·L^−1^) was observed for unidentified cells with a small size at the surface (5 m). This is consistent with a higher 19’HF/TChl*a* ratio usually correlated with high abundance of nanoflagellates [40] and lower diatom-derived pigment concentration (as Chl*c* and fucoxanthine; Table 5); in fact, Chl*c* values were “0” in several samples.

When data were normalized to biovolume, diatoms represented more than 80% of the total large phytoplankton (>10 μm) biovolume at the Coast (87.50% ± 0.90%) and Jet (84.17% ± 3.28%) sites at both depths (surface and DCM) during the sampling period. Total diatom biovolume was significantly lower at the Gyre (5.4 × 10^10^ ± 1.55 × 10^10^ μm^3^·m^−3^) than at the Jet (1.82 × 10^13^ ± 2.53 × 10^13^ μm^3^·m^−3^) or Coast (1.76 × 10^13^ ± 2.51 × 10^13^ μm^3^·m^−3^) sites, with an important decrease in percentage with depth from 74.82% of total large-size phytoplankton biovolume at the surface to 64.93% at the DCM. 

#### 2.4.3. Total Phytoplankton Biovolume Partitioning

As shown in Table 8, the highest percentage of total phytoplankton biovolume referred to diatoms for all sites and both depths (surface and DCM). However, at the DCM, the diatom biovolume was much higher (>80%) at the Coast and Jet sites compared with the Gyre site (58.67%). At the surface, total phytoplankton lacked small-size phytoplankton groups as cyanobacteria at the three sites, with a low average contribution to total biovolume (<1%). At this depth, phytoplankton biovolume was dominated by diatoms and other larger size groups. At the DCM of the three sites, *Prochlorococcus* contributed significantly (>10%) to the total phytoplankton biovolume. This group was especially relevant at the Gyre DCM, with a contribution of 21.61% of the total biovolume. In addition, at the Gyre DCM, dinoflagellates (6.23%) and picoeukaryotes (8.39%) also contributed considerably to the total phytoplankton biovolume. The contribution of the “non-identified” cells (named others) to the total phytoplankton biovolume was not significant (0.19% ± 0.15%) compared with diatoms contribution at all sites and especially at DCM depths (Table 8).

#### 2.4.4. Phytoplankton Physiological State: Quantum Yield (Fv/Fm) and Percentage of Active Chlorophyll (%AChl*a*)

As a general trend, very low values of %AChl*a* and Fv/Fm were obtained for all sites and depths, especially at the Gyre site (Table 9). The highest values of both parameters were observed at the surface and DCM at all stations, with a clear vertical decrease with depth. %AChl*a* never reached values >30%, and it was significantly lower at the Gyre site (one-way ANOVA; *p* < 0.0001) where values showed an average value of 6.05% ± 4.89% throughout the entire sampling period (Table 9). 

### 2.5. Correlations among Polyunsaturated Aldehydes and Physicochemical and Biological Variables

**Figure 6:**
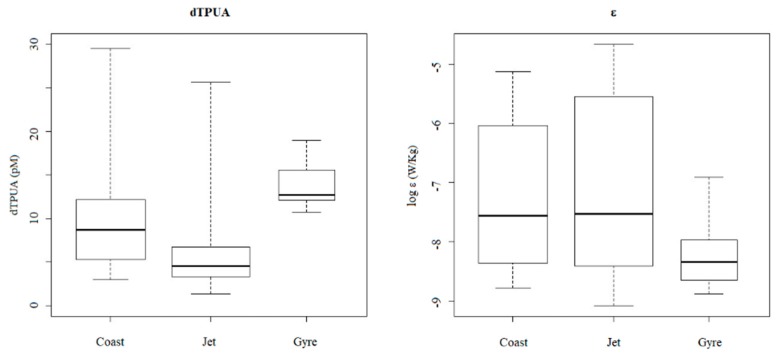
Box plot representing ranges of dissolved total PUA (dTPUA) and rates of dissipation of turbulent kinetic energy (ε) at the different sites.

A Spearman correlation matrix was constructed between dPUA and environmental variables (Table S2, Supplementary Materials). Based on site, significant and positive correlations were established for total dPUA, dC8, and dC10 concentration and ε at the Jet site (Table S2, Supplementary Materials), while no correlation was observed at the Gyre and Coast sites. At shown at Figure 6, total dPUAs were lower at the Jet site, i.e., the station with highest registered values of ε, and higher at the Gyre site (i.e., the station with the lowest ε registered), although this trend was not statistically significant as a consequence of high dispersion with depth as explained above (Figure 6).

Based on percentage of abundance, a significant correlation was established between the relative abundance of dC7 and dC10 and temperature (*p* < 0.05), with a higher percentage of dC7 at lower temperatures and a higher percentage of dC10 at higher temperatures. There were also significant differences in the percentage of abundance of dC10 with photosynthetic active radiation (PAR) (one-way ANOVA; *p*-value = 0.005), obtaining a higher percentage of dC10 at higher irradiances. 

Regarding particulate fractions of PUA, we found positive and significant correlations between total pPUA (pmol from cells in 1 L) diatoms and silicoflagellate abundance (Table S4, Supplementary Materials). By aldehyde, pC7 and pC10 concentration were positive and significantly correlated with diatom and silicoflagellate biovolume (Table S4, Supplementary Materials), while silicoflagellates showed a positive and significant correlation with pC7 and pC8 concentration. Additionally, pC7 and pC8 were positively and significantly correlated with Chl*c*_1_, fucoxanthine, and diadinoxanthine concentrations at the Coast and Jet sites (Table S3, Supplementary Materials).

**Figure 7:**
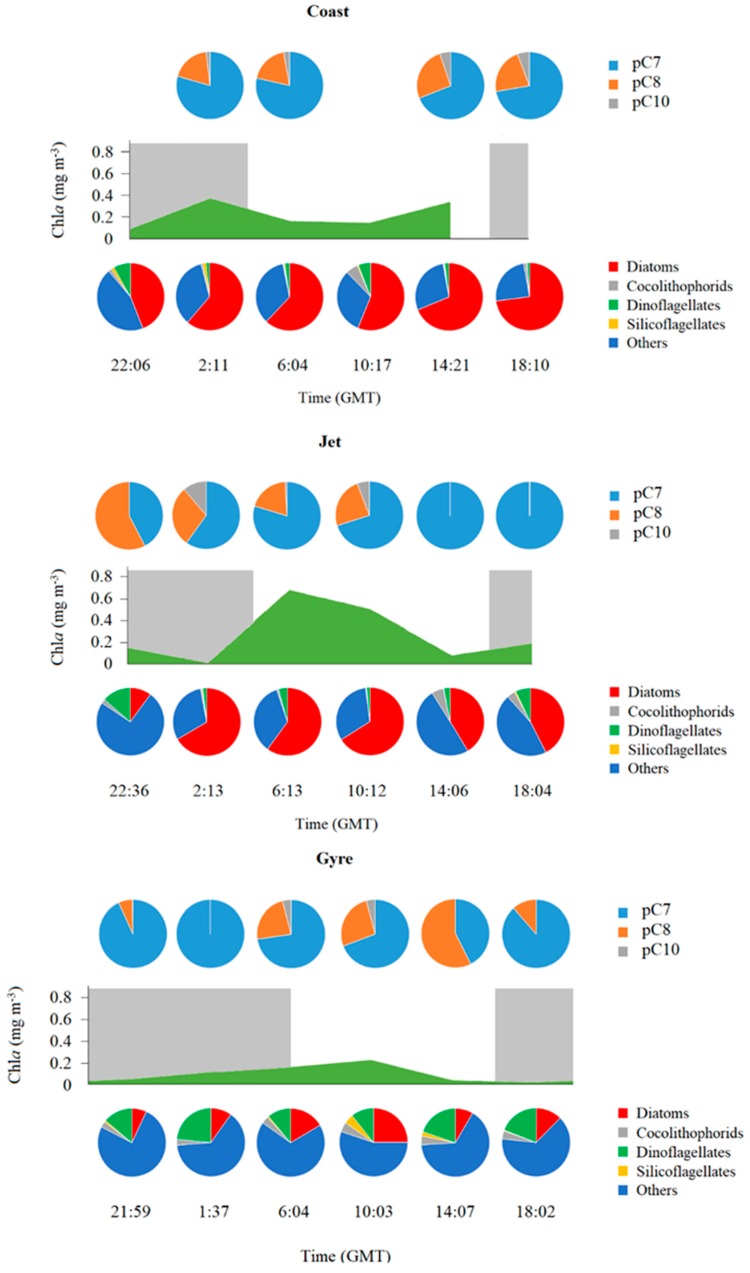
Combined illustration showing the temporal variation along the day of TChl*a* (mg·m^−3^), pPUA (pg·cell^−1^), and large phytoplankton abundance at the DCM of the different sites. Upper pie charts show the percentage of TpPUA of pC7 (2*E*,4*E*/*Z*-heptadienal), pC8 (2*E*,4*E*/*Z*-octadienal), and pC10 (2*E*,4*E*/*Z*-decadienal). Lower pie charts show the percentage of abundance of large-size phytoplankton fraction categories. Gray areas indicate the night period.

Temporal variation along the sampling day of type and concentration of pPUA was not noticeable at the Coast DCM; however, it was clear at the Gyre DCM and Jet DCM (Figure 7). At the Jet DCM, changes in the percentage of abundance of pC7, pC8, and pC10 were accompanied by changes in the percentage of abundance of large-size phytoplanktonic groups (Figure 7). However, this was not observed at the Gyre DCM. 

## 3. Discussion 

The obtained results showed how diatoms dominated the phytoplankton biomass of the three sites (Table 8), decreasing in abundance toward the oligotrophic waters of the Gyre site (Table 7). At the Gyre site, the percentage of cell abundance of unidentified microplankton cells was important (Table 7), as confirmed by a lower DT/Chl*a* ratio (Table 5). However, its contribution to total phytoplankton biomass was lower than that of diatoms at the Gyre surface and not noticeable at the Gyre DCM (Table 8), denoting small-sized cells. At the Gyre DCM, strong oligotrophic conditions favored the increase in abundance of picoplanktonic groups (Table 6) to the detriment of larger microplankton (Table 7). 

The phytoplankton community was physiologically stressed at all sites, a fact evidenced by low values of Fv/Fm and %AChl*a* (Table 9), indicative of non-optimal photosynthetic conditions, especially evident at the Gyre site (Table 9). Reported values of Fv/Fm even for nutrient-replete cyanobacteria in the literature usually range widely from 0.10 to 0.6 [41], and it is known that the effect of initial fluorescence (F_0_) due to phycobiliprotein fluorescence and/or photosystem (PS) I fluorescence contributes to the lower Fv/Fm values observed for many cyanobacteria. Therefore, at the Gyre site, the obtained Fv/Fm values (Table 9) would be in part consequence of a high cyanobacterial abundance (i.e., *Prochlorococcus* and *Synechococcus*). Regarding the large-size phytoplankton, which would also contribute to the measured Fv/Fm and %Achl*a* values, the significant reduction in these parameters at the Gyre, even “0” at several depths, associated with a very low TChl*a* concentration (<0.1 mg·m^−3^) (Table 5) could also be indicative of a high proportion of dead, near dead (chlorotic), or quiescent microplanktonic cells at this site, most likely as a consequence of nutrient starvation. The FlowCAM methodology used for the taxonomical analysis of this phytoplankton large-size fraction cannot discern alive and dead cells, and the visual image analysis is limited for the lower fraction (9–20 μm) [42,43]. It cannot be ruled out that, at the Gyre DCM, persistent cellular structures of dead cells (e.g., frustules, silica rings, coccoliths) could be interpreted, at least in part, as alive cells. In the literature, there is not a clear effect of dead or quiescent phytoplankton cells on the apparent efficiency of PSII (Fv/Fm) [43], but nutrient starvation can be considered as one of the most important causes for phytoplankton cell death in nature [44,45]. Phosphorus limitation could be responsible for such a reduction at the Gyre site, since low phosphatase alkaline activity associated with low phosphorus availability is correlated with low Fv/Fm values of microplankton in nature, and phosphorus limitation was demonstrated to decrease Fv/Fm of several microplanktonic species in culture [46]. Therefore, at the Gyre site, the obtained Fv/Fm values (Table 9) would be a consequence of the coincidence of high cyanobacterial abundance and low nutrient availability. 

Based on the above-explained phytoplankton community description, the pPUA and dPUA quantified in our work were collected in a natural scenario of low resource supply, with a low total phytoplankton biomass (TChl*a* < 1 mg·m^−3^) of highly physiologically stressed cells and with an average relatively high diatom biomass. This represents the opposite situation of a diatom bloom event, where cells have enough resource availability with an optimal physiological state, and total phytoplankton biomass could reach values of TChl*a* > 4–5 mg·m^−3^. In such a scenario, we obtained pPUA from large-size phytoplankton that ranged from 0.02 to 5.3 pmol from cells in 1 L (Figure 3). Compared with most published data [17,25,26,47], these are very low concentrations of pPUA (fg·cell^−1^), i.e., PUA potentially produced by large-size phytoplankton, as well as very low concentrations (pM) of released PUA (dPUA). This range of pPUA is comparable to that previously obtained in subtropical oligotrophic Atlantic areas [48] with low PUA production. The maximum pPUA values per volume were obtained at the Coast DCM, with the highest TChl*a* concentrations (Table 5) and total phytoplankton cell abundance (Table 7). pPUA normalized by cell unit ranged from 0.3 to 70 fg·cell^−1^ with a slight although not significant increase of TPUA per cell, mainly pC7, at the Gyre DCM (Figure 3), the site with the highest N/P ratio (Table 1). This slight increase toward low P-availability conditions is equivalent to that observed by Reference [49] in cultures of *Skeletonema marinoi*. Normalized to TChl*a* concentration, pPUA ranges varied from 0.49 to 9.5 μg TPUA/gChl*a*, consistent with the global trends observed by Reference [30]. In our study, large-size phytoplankton reduced its production of pC10, as the minor aldehyde produced in the low-PUA samples (2.99% ± 4.65% at the Jet, 3.93% ± 1.82% at the Coast, and 1.72% ± 2.23% at the Gyre sites) (Figure 3). These results complement the linkage between PUAs and trophic status defined by Reference [30] since, in this study, we collected large-size phytoplankton with low PUA (limited pC10 production) under low resource supply, a phytoplankton fraction that is usually lost in open ocean oligotrophic areas [30]. The low Fv/Fm and %AChl*a* data obtained (Table 9) corroborate this physiological stress, as a result of limited resource input, particularly of phosphorus, as well as nitrogen at some depths. Phosphorus limitation has an effect on genes encoding fatty acid biosynthesis, which are the main PUA precursors [50,51]. This can strongly suppress PUFA synthesis in large-size phytoplankton cells, as previously experimentally demonstrated for some diatoms such as *Phaeodactylum tricornutum* [51] and *Skeletonema marinoi* reducing the PUFA content [49]. This would limit the available substrate for total oxylipin production for larger phytoplankton. Additionally, N-limited cells may be critical for the production of PUAs, by limiting the synthesis of polypeptides and enzymatic activities [51], increased under low light conditions. This synthesis would most likely be compromised by basal metabolism and, consequently, PUA production would be lower than that expected for a cell preadapted to low resource conditions as in experimental cultures [51]. Ideally, PUFA analysis together with the whole oxylipin (not only PUA) cellular profile, as well as quantification of cellular enzymatic activity, would be desirable for a total comprehension of this effect of combining limiting resources on PUA synthesis for a mixed phytoplankton population in nature, where pulses of nutrients occur, rather than continuous exposure as in experimental cultures.

Diatoms are usually considered as the main large-size phytoplankton group of PUA producers in oceanic waters [8,52]. Other groups, such as haptophytes, also showed the ability to produce PUA in marine environments, e.g., the species *Phaeocystis pouchetii* [53], which produced 2*E*,4*E*-decadienal (C10). In our dataset, the abundance of other phytoplankton groups, such as dinoflagellates and silicoflagellates, were also positively correlated with TpPUA (Table S3, Supplementary Materials). pC7 and pC8 were the most abundant aldehydes quantified for the three sites, as previously described for other oceanic areas [17,27,28,48]. These two aldehydes are usually described as a common combination of aldehydes mainly produced by oceanic diatom species in culture [52], as well as during blooms of *Skeletonema marioni* [25,26], and by natural coastal phytoplankton assemblages [17,27,28]. pC10 was also observed at all sites, although it was less abundant than pC7 and pC8 (Figure 7). Some oceanic diatoms, such as *Thalassiosira rotula, Chaetoceros compressus, Melosira nummuloides*, or *Fragilaria* sp. were also described as C10 producers [52]. At the Gyre site, pC10 was higher than at the Coast and Jet sites (Figure 3b); however, based on the percentage of TpPUA, it was relatively less abundant than pC7 and pC8. These two aldehydes, pC7 and pC8, were positively correlated at the three sites (Table 3), and this could denote an equivalent biological origin, most likely from large diatoms, since, as explained above, most diatoms produce these two aldehydes simultaneously. pC10 followed a different trend and was highly positively correlated with pC8 only at the Gyre site (Table 3), where other phytoplankton groups different from diatoms were also abundant, for which a non-diatom origin for pC10 is plausible.

For the first time, we describe changes of the contribution of each type of aldehyde to the TpPUA for the large-size phytoplankton cells throughout the day. At the Jet site, we found a correlation between the percentage of abundance of pPUA and the percentage of abundance of the large-size phytoplanktonic group. Therefore, the percentage of pC7 was significantly and negatively (Spearman rank correlation; *r* = −0.88; *p* < 0.05) correlated with the percentage of abundance of dinoflagellates, while the percentage of pC8 was positively correlated with it (Spearman rank correlation; *r* = 0.84; *p* < 0.05). We could not find any significant correlation between pPUA partitioning and phytoplankton taxonomical composition at the Coast or Gyre site. However, we found that the percentages of pC7 and pC8 were negatively (Spearman rank correlation *r* = −0.71; *p* < 0.05) and positively (Spearman rank correlation *r* = 0.43; *p* < 0.05) correlated with Fv/Fm and %AChl*a*, respectively, for the whole dataset, denoting a combined effect of species diversity and physiological state on the production of the type of aldehyde; therefore, the variation of pPUA was not strictly correlated with taxonomic diversity but also with cellular physiological state. 

When considering all the PUAs as a set, i.e., TPUA as the sum of pPUA (pmol from cells in 1 L) and dPUA (pM), we found that the contribution of dPUA to the total pool of PUA was higher (≥85%) than that of PUA liberated from wounded cells (pPUA as pmol from cells in 1 L; ≤5%) for the whole dataset (Figure 3). This was also observed for oligotrophic Atlantic waters by Reference [17]. This is indicative that there is more dPUA than PUA that could potentially be produced by lipoxidation of PUFA from large phytoplankton cells after cell breakage during sampling. This trend is contrary to that observed by Reference [25] in the Adriatic Sea, or by Reference [47] in the Pearl River estuary, both strongly eutrophized systems. In the Adriatic Sea, dPUA never reached the values of pPUA and it was 0.5%–1% of pPUA at stations where the well-known PUA producer *Skeletonema marinoi* bloomed. In the Pearl River estuary, dPUA ranged from 0.1–0.37 nM, and values were two orders of magnitude lower than pPUA (0–41 nM). This could be explained by the dPUA fraction including the PUA released by all potential producers, i.e., large- and small-sized phytoplankton cells, while, in this study only, the pPUA from large-size producers was quantified. The contribution of the small-size fraction of phytoplankton is significant, especially in oligotrophic conditions [30]; however, we unfortunately lacked that pPUA fraction. 

Considering the low total phytoplanktonic biomass for the three sites (TChl*a* < 1 mg·m^−3^), the dPUAs quantified in this work are, probably, the lowest level that could be observed at the water column, and this would represent a threshold concentration released after natural processes as cell death/lysis in a low biomass system with high hydrodynamism. These molecules are released after cell wounding, and it is known that phytoplanktonic lysis rates can variate from 0.003 to 1.09 day^−1^, being highest in the most oligotrophic waters [54]. Moreover, phytoplanktonic lysis rates are directly correlated with dPUA concentration, at least in blooming conditions of a PUA producer [55]. It can be assumed that registered dPUAs are not produced immediately at the sampling time. In fact, we probably measured persisting dPUA in the water column for longer periods (days to weeks) than that of our sampling period (24 h). Some results support this interpretation. Firstly, we found higher values of dTPUA that persist longer along the day at the Gyre site (Figure 3C), far below those for the DCM located at 50 m, where lower turbulences were registered (Figure 6). This denotes an effect of turbulence regime in dPUA distribution, as should be expected by its volatile nature. Secondly, the percentage of dC10 was higher at higher temperatures and higher PAR in the detriment of dC7 (Table S2, Supplementary Materials). This trend is consistent with a longer persistence of dC10 as demonstrated by Reference [56] for temperatures of 15 and 20 °C, which are in the range of averaged temperatures measured at the three sites (Table 1). These physical effects of dPUA distribution are indicative of a certain persistence of the aldehydes in the water mass. 

It can be hypothesized that most dPUAs were released back in time, persisting in the water column with the pulsating and continuous addition of a small PUA fraction by cellular lysis rates triggered by resource limitation, especially from large-size phytoplankton, mainly diatoms. This possibility is also supported by the dPUA chemical structure. Most dPUAs were different isomers (Type II), unlike isomers that should be expected from the instantaneous lipoxidation of cellular PUFAs after cell breakage (Type I). These two types of isomers differ in the location of their double bonds along the aldehyde chain. The percentage of Type II isomers of dC7, dC8, and dC10 was much higher than should be expected from the recent release of pC7, pC8, and pC10 (Table 4). Only one publication in the literature analyzed the differences in stability of different polyunsaturated aldehydes in water [57], specifically in drinking water; however, with the exception of Reference [56], there are no data regarding the chemistry of dPUA in seawater or isomer distinction. The presence of different isomers in the dissolved fraction could be a consequence of a different biological origin than pPUA. The small-size phytoplankton could be the source of these Type II isomers quantified as dPUA. However, no positive correlations were established between picoplankton abundance or bacterial abundance (data not shown) and the percentage of total dissolved Type II isomers abundance. Secondly, their presence could also be a consequence of the physicochemical isomerization of the PUA isomers Type I once they are released. Based on the chemical configuration, the isomerization process that would change Type I to Type II dPUA is plausible but cannot occur spontaneously. Type I isomers of dC7, dC8, and dC10 were stable for 10 days in abiotic natural seawater at constant temperatures of 10, 15, and 20 °C in closed agitated systems [56], although the concentration of dC7 and dC8 decreased significantly compared with dC10. In nature, a physical isomerization via photodegradation or trace metal-mediated transformation [58] from Type I to Type II should be considered as a plausible explanation. In our dataset, no correlation was established between the percentage of abundance of Type II isomers and light intensity for any site, although trace metals were not quantified. Thirdly, a biologically mediated process of isomerization could also be assumed. It is well known that the isomerization of polyunsaturated long fatty acids can be mediated by different Gram-negative bacteria [59]. Gram-negative bacteria can adapt to environmental stress by changing their membrane fluidity as a response to rising temperature or the presence of toxic organic compounds by the activity of *cis*- to *trans*-isomerases of unsaturated fatty acids [60]. Although this mechanism is not described as a bacterial or cyanobacterial response to PUA presence, a direct correlation between membrane hydrophobicity and PUA toxicity was observed by Reference [18] in different marine bacterial strains. Bacteria increased the degree of saturation of their membrane fatty acids after exposure to μM levels of PUA. Picoplankton and bacterial abundances significantly and negatively correlated (Spearman rank correlation; *p* < 0.05; *r* = −0.449) with the percentage of abundance of Type II dC7 isomers, the most abundant aldehyde. The PUA Type II isomers could have been generated after the biologically mediated isomerization of released pPUA by free isomerases from dying Gram-negative bacteria (and/or cyanobacteria) in an attempt to protect from its toxicity. Moreover, the free presence of these enzymes in the seawater could be responsible for its isomerization, as it is known that enzymatic activity can persist in the water [61]. In both cases, the presence of these Type II isomers as a part of the dissolved PUA fraction is a consequence of its persistence in the water mass, after the release of a much higher PUA concentration (nM or μM) than that measured (pM). 

The obtained results corroborate the presence of large-size PUA producers in oligotrophic waters with significant diatom biomass. Although the obtained ranges of pPUA (i.e., pM) do not seem to be significant for the copepod–diatom interaction, the main grazers of oligotrophic areas, such as permanent subtropical gyres, are microzooplanktonic species, e.g., ciliates and dinoflagellates, which are known to be much more sensitive to PUA than copepods [13,17] and, thus, effects on these grazers cannot be discarded. Additionally, the concentrations of dPUA were higher than pPUA per volume for the whole dataset. This is the opposite trend to that observed in eutrophized systems or during diatom bloom [25], highlighting the importance of the dPUA fraction in low-resource conditions. These results are coherent with an infochemical function for these molecules, which usually act via direct contact with the cell membrane [18]. In eutrophic systems or bloom events, there is a high cellular density of large-size phytoplankton under non-limiting resources. In such conditions, the probability of cell-to-cell encounters would be higher than in oligotrophic waters, with lower cell densities. During blooms, there is a high resource availability and usually lower cellular lysis rates, which would maintain a constant, but low input of dPUA to the water mass that could trigger any cell-to-cell communication. Contrary to this, in oligotrophic systems, phytoplanktonic lysis rates could reach values > 1.5 day^−1^ [54]. This input of dPUA from phytoplankton would be even higher as a consequence of nutrient-starved dying cells, to the detriment of yjr pPUA fraction, increasing any probability of cell-to-cell communication when low-cell-density conditions would hinder the encounter between cells. Our results also demonstrate the persistence of threshold concentrations of dPUA, whose type and distribution are affected by physicochemical characteristics of the water column, providing evidence of its isomerization in the water column, altering the chemical structure of the released PUA with unknown effects on its stability, biological function, and potential bioactivity. 

## 4. Materials and Methods 

### 4.1. Study Area and Field Sampling

The study area was located in the western Alborán Sea, the most occidental basin of the Mediterranean Sea (southwest Spain) (Figure 1), which is connected to the Gulf of Cádiz through the Strait of Gibraltar. Briefly, the water circulation through the strait is characterized by a two-layer inverse-estuarine circulation: an upper Atlantic layer of nutrient-poor water inflowing as a jet into the Mediterranean Sea, and a deeper and saltier nutrient-rich Mediterranean water outflowing toward the Gulf of Cádiz (see, e.g., Reference [62]). Three water masses are involved in this water exchange through the Strait of Gibraltar: the Surface Atlantic Water (SAW: temperature ~22.6 °C; salinity ~36.4), the North Atlantic Central Water (NACW: temperature 13.6 °C; salinity ~35.8), and the Mediterranean Outflowing Water (MOW: temperature 13.5 °C; salinity ~38.4) [63,64]. The entrance of the Atlantic Jet (AJ) through the Strait is the main driver of the complex circulation of the western Alborán Sea [65,66], characterized by the quasi-permanent Western Alborán Gyre (WAG) [67] and a coastal cyclonic gyre (CsCG) that arises close to the Spanish coast. This CsCG is associated with the upwelling of nutrient-rich waters, mainly influenced by the AJ and the wind regime, standing out for its high productivity [68,69]. A close coupling of mixing processes in the channel, together with transport of coastal phytoplankton-enriched waters toward the Mediterranean sector, was also described as a source of plankton growth and enrichment in the Alborán Sea [70].

On the basis of this known oceanographic description, we selected three sites for sampling: Site 1 was close to the Spanish coast (Coast site), Site 2 was located in the inflowing Atlantic Jet (Jet site), and Site 3 was located in the center of the WAG (Gyre site). These selected sites described a chlorophyll gradient from the coastal to gyre site, as observed in Figure 1. Samples were collected during the MEGAN cruise in the early autumn from 6–9 October 2015, onboard the B/O Sarmiento de Gamboa.

### 4.2. Physicochemical Analysis

Daily sampling was performed at each station every 4 h. Vertical profiles of physicochemical characteristics of the water column were performed using continuous profilers of temperature, salinity, fluorescence, turbidity, and photosynthetic active radiation (PAR), using a Seabird 911 plus CTD device.

Additionally, microturbulence profiles were collected using a vertical microstructure profiler (VMP-250), from Rockland Scientific, sampling at 250 Hz. The microstructure was measured using a tethered free-falling vertical profiler with two high-frequency shear probes, one thermistor, and one conductivity sensor. The rate of dissipation of turbulent kinetic energy (ε, W·kg^−1^) was obtained at the three sites (Coast, Jet, and Gyre) where eight (mean depth 44 m), six (mean depth 154 m), and 10 (mean depth 350 m) vertical profiles were conducted. For comparison between sites, values of ε at different depths were selected. 

Discrete samples were collected at each station every 4 h by Niskin bottles (12 L) mounted on a rosette. Samples at five different depths were selected as a function of physical structure of the water column. At all stations, water was sampled at 5 m and at the deep chlorophyll maximum (DCM). A third sampling depth was selected as a function of the position of the Atlantic–Mediterranean water interface (AMI). Finally, two other complementary and variable sampling depths were selected as a function of station maximum depth (Table 10).

For inorganic nutrient concentration, NO_3_^−^ + NO_2_^−^ (NOx), PO_4_^3−^, and SiO_4_, discrete duplicate samples at selected depths were collected. Filtered seawater (5 mL, 0.7 μm precombusted Whatman GF/F filters) were collected and stored at −20 °C until analysis. Nutrient concentrations were measured at the laboratory using an autoanalyzer (Technicon AA-II-TRACS 800) following the techniques of Reference [71]. 

### 4.3. Biological Variables 

Discrete samples were also used for biological variable quantification. Pigment concentrations, large-size (>10 μm) phytoplankton community analysis, and particulate fraction of PUA (pPUA) from large phytoplankton were analyzed at surface (5 m) and DCM depths. Additionally, dissolved PUA concentration (dPUA), active chlorophyll concentration (%AChl*a*), small-size phytoplankton community analysis, and maximal PSII quantum yield of phytoplankton (Fv/Fm) were analyzed at all sampled depths. 

#### 4.3.1. Pigment Quantification by High-Performance Liquid Chromatography (HPLC)

To obtain total pigments, 1 L of water was collected at 5 m and DCM depths, and filtered through GF/F filters (0.7 μm Whatman). All filters were stored in liquid nitrogen during the survey and, once at the laboratory, stored at −80°C. Pigments were extracted using 99.9% pure acetone for 24 hours at 4 °C. Analysis of samples was performed using high-performance liquid chromatography (HPLC Waters Thermofisher) following the method of Reference [72]. Concentrations of chlorophyll *c*1, *c*2, and *c*3, peridinin, 19’-butanoyloxyfucoxanthin, fucoxanthin, violaxanthin, prasinoxanthin, micromonal, 19’-hexanoyloxyfucoxanthin, diadinoxanthin, alloxanthin, diatoxanthin, lutein, chlorophyll *b*, and chlorophyll *a* were later calculated using the area of the peak and retention time for each one. With the aim of a better pigment separation, 10% of Milli Q water was added to the extract at the time of the analysis [72].

#### 4.3.2. Calculation of Trophic Index (Fp)

A trophic index (Fp) was defined as the ratio of the integrated concentration of fucoxanthine and peridinine. Both pigments were quantified by high-performance liquid chromatography (HPLC) as the sum of the integrated concentration of diagnostic pigments of all eukaryotic taxa that may be present in a phytoplankton community (modified from Reference [35]) excluding zeaxanthine and divinyl Chl*b* indicative of prokaryote taxa.

Fp = [Fucoxanthine + Peridinine] × [Fucoxanthine + Peridinine + 19’HF + 19’BF + Chlb + Alloxanthine]^−1^.

The diagnostic pigments used to characterize the main oceanic phytoplankton groups were as follows: fucoxanthine for diatoms, peridinine for dinoflagellates [73], 19’-hexanoyloxyfucoxantine (19’HF) and 19’-butanoyloxyfucoxanthine (19’-BF) for nanoflagellates as prymnesiophytes [74], chlorophyll *b* for green flagellates [73], and alloxanthine for cryptophytes [75].

Furthermore, the index DT/TChl*a* was calculated as follows:

DT/Chl*a* = [∑[Chl*c* (Chlc_1_,c_2_,c_3_), fucoxanthine, diatoxanthine,diadinoxanthine]] × [Chl*a*]^−1^.

This ratio was used to check consistencies between phytoplankton community analysis by Flow-CAM and HPLC pigment quantification.

#### 4.3.3. Active Chlorophyll and Maximal PSII Quantum Yield 

As indicators of phytoplankton physiological state, two photosynthetic parameters were analyzed during the sampling period: percentage of active chlorophyll *a* (%AChl*a*) and maximal PSII quantum yield (Fv/Fm). These parameters were measured using a pulse amplitude modulated (PAM) fluorometer specifically designed to study phytoplankton cells (PhytoPAM^®^, Heinz Walz GmbH, Effeltrich, Germany). The PhytoPAM uses weak probe flashes to measure the change in the quantum yield of fluorescence induced by a strong pump flash. This relative change is proportional to the quantity of chlorophyll included in active photosystem II (PS II) reaction centers, as successive light pulses lead to a saturation of PSII centers and a diminution of the fluorescence quantum yield. Thus, the PhytoPAM provides an estimate of the proportion of total chlorophyll within active PSII, i.e., the chlorophyll available for photosynthesis [76] or “active chlorophyll”. The maximal PSII quantum yield measurements were done onboard with 15-min dark-adapted seawater samples from each station and depth. Both parameters were measured for fractionated (< 10 μm) and total phytoplankton fraction. PAM fluorimeters, such as that used in the present study, use white excitation light that excites PSII effectively and should, thus, minimize the tendency to underestimate the true quantum efficiency of Cyanobacteria. We ensured that Fv/Fm was not underestimated by quantifying Fv/Fm in the lab using nutrient-replete cultures of *Synechococcus*; we observed Fv/Fm in the range 0.28–0.49 with this same PAM.

#### 4.3.4. Phytoplankton Community Analyses 

Phytoplankton cell abundances and total biovolume were analyzed via different methodologies. To cover the identification of all planktonic groups, two different techniques were used for the community analyses. Small-size phytoplanktonic groups (< 10 μm) were analyzed by flow cytometry as explained below. Large-size phytoplanktonic fractions (10–250 μm) were analyzed by FlowCAM^®^ (Fluid Imaging Technologies). 

##### Small-Size Phytoplankton Analyses by Flow Cytometry 

Cytometry samples were collected at every depth during the daily cycle at each site. Firstly, 3.6 mL of seawater was collected and fixed with 0.5% glutaraldehyde/0.05% formaldehyde and analyzed onboard using a flow cytometer (Becton-Dickinson FacsAria). Four groups were distinguished in the obtained cytograms: *Prochlorococcus, Synechococcus*, Nanoeukariotes, and Picoeukariotes (see Figure S1, Supplementary Materials, showing a typical cytogram). Cytometer settings were established performing a previous calibration with natural algae cultures from the microalgae collection of the Instituto de Ciencias Marinas de Andalucía (ICMAN-CSIC). Heterotrophic bacteria were determined by dying 0.5 mL of the fixed cytometry samples described above with 2.5 µL of SYTO 13 (Green Fluorescent Nucleic Acid Stain, Thermofisher) for 10 min in darkness. Two bacterial populations were determined during analysis: a high-DNA-content population (HNA), considering that with a higher SYTO 13 signal, and a low-DNA-content (LNA) population with a lower SYTO 13 signal [77].

##### Large-Size Phytoplankton Analysis (10–250 μm) by Fluid Imaging FlowCAM^®^

Two size fractions were analyzed: 10–100 μm and 100–250 μm. For the 10–100-μm fraction, 5-L samples were collected at 5 m and DCM depths and passed through two consecutive meshes of 100 μm (to discard larger organisms) and 10 μm mesh. Organisms retained at this second mesh were reconcentrated with 0.7-μm-filtered seawater to a final volume of 125 mL. This size fraction (10–100 μm) was analyzed in vivo onboard after water sampling using a FlowCAM to count and measure all organisms. For the larger size fraction (100–250 μm), 10 L was passed through two consecutive meshes of 250 and 100 μm, and concentrated to a final volume of 100 mL, discarding organisms larger than 250 μm. This fraction was fixed with buffered formaldehyde (4% final concentration), stored in amber glass flasks, and later analyzed in the laboratory. For both fractions FlowCAM was used in auto-trigger mode [78], with a chamber of 2.0 mm × 0.1 mm and a 10× objective for the small fraction, and a chamber of 3.0 mm × 0.3 mm and a 4× objective for the larger fraction. In both cases, the whole sample was completely processed. The obtained images were sorted automatically using a previously trained neural network KERAS (https://keras.io/), which separates the particles into 31 groups of living particles and three groups of non-living particles. From a taxonomic point of view, these groups are merged into five “functional classes” (diatoms, flagellates, dinoflagellates, ciliates, and other unidentified living particles). A detailed description of differential categories is available in Table S1 (Supplementary Materials). The functional classes of diatoms were studied in much more detail as the main PUA producers. Each category was associated with a simple geometrical shape; thus, the individual cell biovolume was calculated using the dimensions (i.e., diameter, length and width) measured by the FlowCAM software. A previous volume correction was performed for fixed samples (100–250 μm) [79]. 

#### 4.3.5. Sampling and Extraction of Polyunsaturated Aldehydes (pPUA and dPUA)

Samples for the quantification of pPUA from large-size phytoplankton (>10 μm) were collected at surface and DCM depths of each sampling station every 4 h, using 25 L of seawater. This volume was passed through two consecutive meshes of 250 μm and 10 μm, to collect the large-size phytoplankton fraction covering the same size range as determined by FlowCAM analysis (10–250 μm). The phytoplankton retained in the 10-μm mesh was diluted in 125 mL of filtered seawater and then passed through a nucleopore polycarbonate filter with a 0.4-μm pore size (GE PoreticsTM). This filter was then transferred to a 25-mL glass vial (Teknokroma) and rinsed using 1 mL of 25 mM *O*-(2,3,4,5,6-pentafluorobenzyl) hydroxylamine hydrochloride (PFBHA), derivatization grade >98% (Sigma-Aldrich, Switzerland), in Tris-HCl 100 mM at pH 7.2 (Trizma, Sigma). This reagent stabilizes aldehydes via derivatization, preventing volatilization and eventual transformation before analysis using gas chromatography/mass spectrometry (GC–MS). These samples were then stored at −20 °C until extraction onto a hexane/methanol/water (2:1:2) funnel after mechanical cell disruption, following the same protocol as Reference [27]. 

Samples for dPUA analysis were obtained at all sites and sampling depths using 1 L of natural seawater. This volume was filtered onboard at very low vacuum pressure (<500 mbar) through a Whatman ^®^ glass microfiber filter grade GF/F. Then, 1 mL of PFBHA and benzaldehyde as standard (at a final concentration of 5 nM) was added. Bottles were maintained at ambient temperature and in the dark for at least 1 h to ensure total derivatization of dPUA. Samples were then passed through a LiChrolut^®^ RP 18 cartridge previously washed with derivatization solution, with a flow rate of 0.8–1.0 L·h^−1^. The derivatized PUAs were eluted from the cartridge with 4 mL of 10 mM PFBHA in methanol, collected in a glass vial, and incubated for at least 1 h at ambient temperature to ensure complete derivatization of the aldehydes. Vials were then stored at −20 °C until extraction at the lab onto a hexane/methanol/water (2:1:2) funnel following the protocol of Reference [56].

#### 4.3.6. pPUA and dPUA Quantification

Analysis of extracts was conducted using an Agilent 7890A GC system (Agilent Technologies Inc., Santa Clara, CA, USA) coupled to a high-resolution mass spectrometer Waters Synapt G2 Q-TOF (Milford, MA, USA) equipped with an Atmospheric Pressure Gas Chromatography (APGC) ionization source. Capillary gas chromatography separation of PUAs was performed using an HP-5MS column (30 m × 0.25 mm inner diameter (i.d.) × 0.25 mm film thickness consisting of 5% phenyl and 95% polydimethylsiloxane), keeping the helium carrier gas flow at 1 mL·min^−1^ and the injection port temperature at 280 °C. The column temperature ramp was as follows: 70 °C for 1 min, increased by 35 °C·min^−1^ to 180 °C, then by 4.50 °C·min^−1^ to 290 °C, and held for 8 min. Time-of-flight mass spectrometry was used for the identification and quantification of analytes (Waters Synapt G2). Atmospheric Pressure Ionization(API) positive polarity mode was selected. The mass range considered was *m*/*z* = 50–1200. Corona voltage was 2 kV, and the source temperature was 130 °C. Different sampling cone voltages (from 10 to 40 V) were tested. 

Identification of analytes was based on comparing retention times and accurate mass measurements (allowing an error of less than 5 ppm) to those for commercially available pure standards, 2*E*,4*E*-heptadienal (90%, Sigma-Aldrich Chemie GmbH, Steinheim, Germany), 2*E*,4*E*-octadienal (≥96%, Sigma-Aldrich Chemie GmbH), and 2*E*,4*E*-decadienal (85%, Sigma-Aldrich Chemie GmbH). Quantification of target compounds was performed using calibration curves (from 1 to 7000 nM, prepared in hexane 2-mL vials, and taking into account the signal intensities of the standards at the pseudomolecular ions. The results were obtained by plotting the peak area of each aldehyde [M + H]^+^, 306.0912 for C7 derivatives, 320.1064 for C8 derivatives, and 348.1372 for C10 derivatives relative to the internal standard (benzaldehyde, [M + H]^+^, 302.0609). The reproducibility and repeatability of the methods were evaluated by performing three successive extractions and injections of the sample and by re-analyzing a batch of standards two weeks after the first analysis. Chromatograms were evaluated with the MassLynx software (version 4.1, Waters, Milford, MA, USA). 

### 4.4. Data Analysis

The maps were generated using Ocean Data View. Statistical analysis was performed using statistical package *R*. Data were checked for normality and homogeneity of variance prior to analysis. Analyses of variance (ANOVAs) were performed on the pPUA and dPUA between sites or depths (5 m and DCM). The Kruskal–Wallis rank test and pos hoc pairwise comparison by means of the Mann–Whitney test were applied for comparing physicochemical parameters at different sites. Spearman correlation analyses were performed both environmental (temperature, salinity, photosynthetic active radiation, nitrate, phosphate, silicate, epsilon) and biological data (*p*PUA, *d*PUA, pigments, and phytoplankton composition). 

## 5. Conclusions

Our results corroborate the presence of large-size PUA producers in oligotrophic waters with significant diatom biomass. For the first time, we observed a combined effect of phytoplankton species diversity and physiological state on the production of the type of aldehyde. We also found that threshold concentrations of dPUA are persistent in the water column, with the type and distribution affected by physicochemical characteristics of the water column. Our results provide evidence of the isomerization of dPUA in the water column, altering the chemical structure of the released PUA. 

## Figures and Tables

**Table 1:** Vertical averaged physicochemical characteristics of the sampling sites. Fp: trophic index. NO_x_ = (NO_3_^−^ + NO_2_^−^). See methods for a detailed explanation of its calculation. Data are expressed as average ± standard deviation and [maximum–minimum] of the water column.

	Coast (0–50 m)	Jet (0–200 m)	Gyre (0–400 m)
Trophic index (Fp)	0.77 ± 0.07 [0.9–0.63]	0.073 ± 0.095 [0.28–0.01]	0.13 ± 0.14 [0.44–0]
Temperature (°C)	16.31 ± 1.17 [14.08–17.74]	16.56 ± 2.18 [13.33–20.75]	18.75 ± 4.16 [13.37–23.27]
Salinity	37.06 ± 0.33 [36.56–37.77]	36.69 ± 0.75 [36.22–38.27]	37.11±0.79[36.43–38.5]
NOx (μM)	2.78 ± 1.79 [6.1–0.3]	2.94 ± 2.32 [7.83–0.17]	2.74 ± 3.52 [9.89–0.05]
PO_4_^3−^ (μM)	0.22 ± 0.11 [0.34–0.01]	0.35 ± 0.43 [2.42–0]	0.16 ± 0.16 [0.45–0]
SiO_4_ (μM)	2.99 ± 1.39 [7.9–1.4]	2.68 ± 1.97 [9.39–0.62]	2.27 ± 2.26 [7.02–0.15]
NOx/PO_4_^3−^	17.8 ± 16.2 [56.8–1.69]	10.71 ± 7.57 [24.96–1.02]	25.51 ± 39.45 [175–0.6]
SiO_4_/NOx	1.76 ± 1.57 [7.18–0.72]	2.3 ± 3.7 [16.80–0.45]	3.99 ± 6.94 [37.7–0.43]

**Table 2:** Recorded data of the rate of dissipation of turbulent kinetic energy (ε, m^2·^s^−3^) at the three sites. Data are expressed as mean ± standard deviation and [maximum–minimum]. Results of the analysis of two-way ANOVA showing the effects of site and depth (5–50 m) and their interactions on ε; df—degrees of freedom; MS—mean squares.

Epsilon, ε (m^2^ s^−3^)	Coast	Jet	Gyre	
**Depth averaged** **(5–200 m)**	1.06 10^−6^ ± 2.07 10^−6^ [1.63 10^−9^–7.44 10^−6^]	3.25 10^−6^–6.05 10^−6^ [8.24 10^−10^ ± 2.18 10^−5^]	1.71 10^−8^ ± 3.43 10^−8^ [1.32 10^−9^–1.22 10^−7^]	
**Daily averaged** **(5 m)**	3.16 10^−6^ ± 2.6 10^−6^	1.21 10^−5^ ± 5.8 10^−6^	-	
**Daily averaged** **(25 m)**	1.12 10^−8^ ± 1.25 10^−8^	1.84 10^−6^ ± 3.65 10^−6^	6.032 10^−9^ ± 6.54 10^−9^	
**Source of variation**	**df**	**MS**	**F**	***p***
**site**	2	6.37 10^−11^	4.835	0.017
**Depth**	1	3.33 10^−10^	25.27	0.00039
**Site × depth**	1	2.89 10^−10^	21.99	0.000091

**Table 3:** Spearman rank correlation matrix among polyunsaturated aldehydes for the whole dataset of the particulate (pPUA; pg·cell^−1^) and dissolved (dPUA; pM) fraction of polyunsaturated aldehydes. * significant at *p* < 0.01.

	**pC7**	**pC8**	**pC10**
pC7	1		
pC8	0.69 *	1	
pC10	0.49	0.76 *	1
	dC7	dC8	dC10
dC7	1		
dC8	0.85 *	1	
dC10	0.25	0.39 *	1

**Table 4:** Percentage of abundance of observed types of isomers of particulate PUA (pPUA) and dissolved PUA (dPUA): Type I and Type II (see methods section for details). Data are averaged for 5 m and DCM for pPUA and averaged for the entire water column for dPUA. C7: 2*E*,4*E*/*Z*-heptadienal. C8: 2*E*,4*E*/*Z*-octadienal; C10: 2*E*,4*E*/*Z*-decadienal.

	**pC7-Type I**	**pC7-Type II**	**pC8-Type I**	**pC8-Type II**	**pC10-Type I**	**pC10-Type II**
**Coast 5 m**	98.21 ± 1.11	1.79 ± 1.11	55.83 ± 11.82	44.17 ± 11.82	44.73 ± 17.45	55.27 ± 17.45
**Coast DCM**	98.72 ± 0.73	1.28 ± 0.73	63.45 ± 9.21	36.55 ± 9.21	32.91 ± 4.73	67.09 ± 4.73
**Jet 5 m**	99.20 ± 0.65	0.8 ± 0.65	17.59 ± 11.57	82.41 ± 11.57	73.69 ± 22.72	26.31 ± 22.72
**Jet DCM**	85.86 ± 31.40	1.11 ± 1.03	21.55 ± 11.73	80.83 ± 11.55	53.34 ± 28.31	46.66 ± 28.31
**Gyre 5 m**	92.27 ± 6.73	7.73 ± 6.73	25.51 ± 20.25	74.49 ± 20.25	58.64 ± 15	41.36 ± 15
**Gyre DCM**	98.57 ± 1.23	1.43 ± 1.23	14.24 ± 24.60	85.76 ± 24.60	65.51 ± 20.32	34.49 ± 20.32
	**dC7-Type I**	**dC7-Type II**	**dC8-Type I**	**dC8-Type II**	**dC10-Type I**	**dC10-Type II**
**Coast**	8.66 ± 4.92	91.34 ± 4.92	4.28 ± 2.14	95.72 ± 2.14	2.08 ± 4.42	97.92 ± 4.42
**Jet**	7.82 ± 4.13	92.18 ± 4.13	3.59 ± 1.47	96.41 ± 1.47	1.56 ± 0.89	98.44 ± 0.89
**Gyre**	3.32 ± 2.19	96.68 ± 2.19	5.41 ± 1.98	94.59 ± 1.98	0.90 ± 0.35	99.10 ± 0.35

**Table 5:** Daily average pigments concentrations (mg·m^−3^) at 5 m and DCM depths at the different sites. 19’hex: 19’-hexanoyloxyfucoxanthin; 19’but: 19’-butanoyloxyfucoxanthin. Ratio DT/Chl*a* = [∑[Chl*c* (Chl*c*_1_,*c*_2_,*c*_3_), fucoxanthine, diatoxanthine,diadinoxanthine]] × [Chl*a*]^−1^. DT denotes diatom.

	Coast		Jet		Gyre	
*Depth*	*5 m*	*DCM*	*5 m*	*DCM*	*5 m*	*DCM*
**Chlorophyll *a***	0.311 ± 0.173	0.209 ± 0.125	0.220 ± 0.128	0.312 ± 0.260	0.047 ± 0.039	0.087 ± 0.079
**Chlorphyll *b***	0.021 ± 0.005	0.011 ± 0.004	0.015 ± 0.007	0.032 ± 0.009	0.003 ± 0.002	0.029 ± 0.014
**Chlorophyll c**	0.054 ± 0.049	0.042 ± 0.049	0.017 ± 0.018	0.026 ± 0.027	0	0.002 ± 0.004
**Peridinine**	0.007 ± 0.013	0.002 ± 0.002	0.005 ± 0.006	0.002 ± 0.003	0.003 ± 0.003	0.003 ± 0.004
**19’hex + 19’but**	0.0980 ± 0.0495	0.055 ± 0.062	0.087 ± 0.037	0.166 ± 0.050	0.038 ± 0.020	0.121 ± 0.060
**Ratio DT/Chl*a***	1.680 ± 0.209	1.870 ± 0.645	0.951 ± 0.376	0.859 ± 0.163	2.287 ± 2.289	0.687 ± 0.527
**Ratio 19’hex/Chl*a***	0.262 ± 0.065	0.224 ± 0.138	0.394 ± 0.194	0.587 ± 0.323	1.769 ± 1.669	1.948 ± 2.406

**Table 6:** Daily average cell abundance of small-size phytoplanktonic groups (<10 μm) (SPhA; cell·L^−1^) and total large-size phytoplankton (10–250 μm) (LPhA; cell·L^−1^) at the surface and DCM at different sites. The daily averaged biovolume of both groups (SPhB and LPhB; μm^3^·L^−1^) is also detailed.

	Coast		Jet		Gyre	
Depth	5 m	DCM	5 m	DCM	5 m	DCM
SPhA	2493 × 10^3^ ± 602.5 × 10^3^	2105 × 10^3^ ± 841.15 × 10^3^	2571 × 10^3^ ± 863 × 10^3^	3068 × 10^3^ ± 1649 × 10^3^	710 × 10^3^ ± 198.2 × 10^3^	4451 × 10^3^ ± 1180 × 10^3^
*Synechococcus*	538 × 10^3^ ± 206 × 10^3^	438 × 10^3^ ± 186 × 10^3^	1170 × 10^3^ ± 349 × 10^3^	575 × 10^3^ ± 174 × 10^3^	501 × 10^3^ ± 139 × 10^3^	393 × 10^3^ ± 188 × 10^3^
*Prochlorococcus*	414 × 10^3^ ± 62.4 × 10^3^	447 × 10^3^ ± 55.7 × 10^3^	555 × 10^3^ ± 184 × 10^3^	1670 × 10^3^ ± 177 × 10^3^	113 × 10^3^ ± 59 × 10^3^	3880 × 10^3^ ± 989 × 10^3^
Picoeukaryotes	1361 × 10^3^ ± 472 × 10^3^	1065 × 10^3^ ± 602.1 × 10^3^	773 × 10^3^ ± 568 × 10^3^	736 × 10^3^ ± 446 10^3^	80 × 10^3^ ± 18 × 10^3^	37.2 × 10^3^ ± 22.5 × 10^3^
Nanoeukaryotes	180.2 × 10^3^ ±129 × 10^3^	155.3 × 10^3^ ± 149.2 × 10^3^	73.5 × 10^3^ ± 22.8 × 10^3^	82.9 × 10^3^ ± 4402 × 10^3^	14.9 × 10^3^ ±10.1 × 10^3^	22.49 × 10^3^ ± 12.32 × 10^3^
SPhB	1.38 10^7^ ± 2.78 10^6^	7.34 × 10^6^ ± 6.87 × 10^6^	1.27 × 10^7^± 4.52 × 10^6^	8.18 × 10^6^ ± 4.41 × 10^6^	2.57 × 10^6^ ± 6.73 × 10^5^	1.06 × 10^7^ ± 3.64 × 10^5^
LPhB	2.7 × 10^12^ ± 2.64 × 10^12^	1.53 × 10^8^ ± 6.1 × 10^7^	1.66 × 10^12^ ± 1.94 × 10 ^12^	4.4 × 10^9^ ± 3.32 × 10^9^	1.58 × 10^11^ ± 1.12 × 10^11^	1.0 × 10^9^ ± 6.76 × 10^8^
LPhA	46.6 × 10^3^ ± 36.7 × 10^3^	29.8 × 10^3^ ± 34.3 × 10^3^	11.1 × 10^3^ ± 7.1 × 10^3^	12.9 × 10^3^ ± 9.78 × 10^3^	4.86 × 10^3^ ± 3.99 × 10^3^	3.03 × 10^3^ ± 2.03 × 10^3^

**Table 7:** Cell abundance expressed as daily percentage of total abundance of large-size (10–250 μm) phytoplankton fraction at 5 m and DCM of the different sites. Taxonomical categories are detailed in Table S1 (Supplementary Materials).

	Diatoms	Others	Coccolitophorids	Silicoflagellates	Dinoflagellates
**Coast 5 m**	62.12 ± 9.73	33.12 ± 5.15	1.07 ± 0.88	0.54 ± 0.57	3.13 ± 4.20
**Coast DCM**	60.88 ± 10.13	33.07 ± 6.99	1.73 ± 2.04	0.84 ± 0.57	3.48 ± 2.85
**Jet 5 m**	45.03 ±16.02	46.15 ± 11.84	1.83 ± 1.57	0.59 ± 0.53	6.41 ± 3.85
**Jet DCM**	47.77 ± 21.58	44.49 ± 16.52	2.22 ± 2.15	0.32 ± 0.26	5.21 ± 4.59
**Gyre 5 m**	21.59 ± 11.19	59.11 ± 11.84	3.69 ± 2.12	0.52 ± 0.77	15.09 ± 4.48
**Gyre DCM**	13.18 ± 6.68	65.46 ± 6.65	3.58 ± 0.73	1.45 ± 1.68	16.33 ± 5.23

**Table 8:** Total phytoplankton biovolume partitioning expressed as daily averaged percentage of total biovolume at the surface (5 m) and DCM of the different sites. Taxonomical categories are detailed in Table S1 (Supplementary Materials).

	Diatoms	Coccolitophorids	Silicoflagellates	Dinoflagellates	*Prochlorococcus*	*Synechococcus*	Picoeukaryotes	Nanoeukaryotes	Others
**Coast 5 m**	83.37 ± 2.98	0.49 ± 0.28	0.12 ± 0.15	0.97 ± 0.60	0.097 ± 0.12	0.17 ± 0.22	0.21 ± 0.18	1.34 ± 1.61	13.6 ± 0.43
**Coast DCM**	86.61 ± 10.58	0.09 ± 0.02	0.05 ± 0.04	0.35 ± 0.24	11.16 ± 8.77	0.43 ± 0.70	0.12 ± 0.16	0.43 ± 0.70	0.08 ± 0.06
**Jet 5 m**	88.30 ±1.58	0.39 ± 0.32	0.03 ± 0.02	1.75 ± 0.86	0.12 ± 0.08	0.48 ± 0.36	0.26 ± 0.29	0.48 ± 0.20	8.30 ± 2.28
**Jet DCM**	80.41 ± 10.30	0.27 ± 0.34	0.01 ± 0.01	1.74 ± 2.56	14.45 ± 7.67	0.22 ± 0.32	1.19 ± 2.30	0.22 ± 0.32	0.17 ± 0.15
**Gyre 5 m**	84.88 ± 0.81	0.14 ± 0.01	0.20 ± 0.28	3.01 ± 0.22	0.12 ± 0.01	0.7 8 ± 0.28	0.13 ± 0.08	0.61 ± 0.05	9.91 ± 0.23
**Gyre DCM**	58.65 ± 13.69	1.19 ± 0.98	0.48 ± 0.03	6.23 ± 4.99	21.61 ± 11.71	1.28 ± 0.45	8.39 ± 3.12	1.28 ± 0.47	0.37± 0.14

**Table 9:** Average ± standard deviation and maximum values registered for percentage of active chlorophyll (%AChl*a*) and quantum yield (Fv/Fm) for total phytoplankton at the three sites. Maximum values were observed at 5 m (*) or DCM (**).

	Coast	Jet	Gyre
%AChl*a*	15.35 ± 7.80 [31.25**]	17.92 ± 19.22 [91.07 **]	6.05 ± 4.89 [13.06*]
Fv/Fm	0.23 ± 0.14 [0.46*]	0.11 ± 0.14 [0.48*]	0.036 ± 0.08 [0.26**]

Regarding Fv/Fm, there was a general decrease from surface to 50 m. This decrease was especially patent at the Jet site. At the Gyre site, Fv/Fm was significantly lower compared with the other two sites (one-way ANOVA; *p* < 0.001) throughout the sampling period with an average value of 0.036 ± 0.08 (Table 9).

**Table 10:** Site coordinates and sampling depths (m). DCM: deep chlorophyll maximum; AMI: Atlantic–Mediterranean interface. DCM and AMI depth varied every 4 h during the sampling day. N—north; W—west.

Sites	Coordinates	Surface	DCM	AMI	Complementary	Floor
Coast	36.398° N–5.156° W	5	10, 12, 19	-	20, 25	50
Jet	35.998° N–5.129° W	5	17, 25, 40	50	75	200
Gyre	35.705° N–4.405° W	5	50	65, 68, 70, 75, 80	175, 220	400

## Supplementary Materials

The following are available online at https://www.mdpi.com/1660-3397/18/3/159/s1: Figure S1. Molecular structure of isomers Type I; Table S1. Phytoplankton functional groups used for FlowCAM quantification of large-size phytoplankton; Table S2. Spearman rank correlation coefficients performed on particulate (pPUA) and dissolved PUA (dPUA) data and environmental variables; Table S3. Spearman rank correlation coefficients performed on pPUA data and biological variables I (pigments); Table S4. Spearman rank correlation coefficients performed on pPUA data and biological variables II (biovolume of taxonomical categories of large-size phytoplankton).
