# Supplementary material for: Types and Distribution of Bioactive Polyunsaturated Aldehydes in a Gradient from Mesotrophic to Oligotrophic Waters in the Alborán Sea (Western Mediterranean)
Source: Mar Drugs. 2020 Mar 12;18(3):159. doi: 10.3390/md18030159 (PMC7143741; doi:10.3390/md18030159)
Supplement: Supplementary file 1 [file marinedrugs-18-00159-s001.pdf]

Supplementary Figures

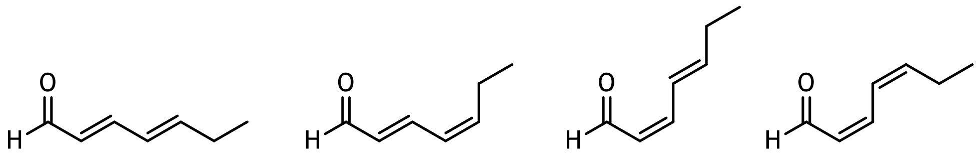

(*E*,*E*) (*E*,*Z*) (*Z*,*E*) (*Z*,*Z*)

Figure S1: Molecular structure of isomers Type I using heptadienal as an example.

Supplementary tables

Table S1: Phytoplankton functional groups used for FLOW CAM quantification of large size phytoplankton (10-250 µm) cell abundance and biovolume. Grouping name for analysis is detailed.

Category	Size range 09 to 20 µm	Size range 20 to 100 µm	Size range 100 to 250 µm
**Others**	Spherical cells	Spherical cells	
	Elliptical cells	Elliptical cells	Elliptical cells
	Discoid cells	Discoid cells	
	Cylindrical cells		
	Filamentous chains	Filamentous chains	Filamentous chains
**Coccolithophorids**		Coccolithophores	
**Silicoflagellates**	Silicoflagellates	Silicoflagellates	Silicoflagellates
**Dinoflagellates**		*Protoperidinium*	*Protoperidinium*
		Ceratium	*Ceratium*
		Prorocentrum	*Prorocentrum*
		Gymnodiniales	*Gymnodiniales*
		Peridiniales	
		Unidentified dinoflagellates	Unidentified dinoflagellates
**Tintinids**		Extended-lorica tintinnids	Extended-lorica tintinnids
		Globular-lorica tintinnids	Globular-lorica tintinnids
**Diatoms**		Pennate diatoms	Pennate diatoms
		Needle-like diatoms	Needle-like diatoms
		Rhizosolenia & *Proboscia*	Proboscia
		Wide-chain diatoms	Pleurosigma
		Guinardia striata	
		Blavyanus flaccida	
		Leptocylindrus	
		Filamentous diatoms	
		Slightly-connected diatoms	
		Chaetoceros	*Chaetoceros*
		Skeletonema	*Skeletonema*
		Unidentified single centric diatoms	Unidentified single centric diatoms
		Unidentified short-chain diatoms	
		Unidentified long-chain diatoms	
		Thalassiosira	
		Asterionellopsis	*Asterionellopsis*
		Thalassionema	*Thalassionema*
Phaeocystis		*Phaeocystis*	

Table S2: Spearman rank correlation coefficients performed on particulate (pPUA) and dissolved PUA (dPUA) data and enviromental variables. d: dissolved; p: particulate; C7: 2*E*,4*E*/*Z*-heptadienal. C8: 2*E*,4*E*/*Z*-octadienal; C10: 2*E*,4*E*/*Z*-decadienal; T: Temperature (ºC); S: Salinity; PAR: Photosynthetic Active Radiation (mmolquanta m^{-2} s^{-1}); F: Fluorescence (f.u.); ε: coefficient of dissipation of turbulent kinetic energy (m^2 s^{-3}); Nitrate, phosphate and Silicate: μM. O_2: mg L^{-1}. * significant at level 0.05: ** significant at level 0.01.

Station depth	PUA	T	S	PAR	F	NO_3^-	PO_4^{3-}	SiO_4	ε	Oxygen
Jet 5 m	**dPUA**	0.206	-0.181	0.034	0.180	-0.140	-0.058	-0.186	**0.598****	0.198
	**dC7**	0.259	-0.169	0.08	0.136	-0.195	-0.028	-0.162	0.333	0.227
	**dC8**	0.182	-0.204	-0.022	0.200	-0.137	-0.074	-0.206	**0.660****	0.193
	**dC10**	0.169	-0.119	0.072	0.133	-0.094	-0.039	0.068	**0.522****	0.15
	**pPUA**	**0.55**	**0.67**	-0.458	**0.748**	-0.317	0.189	-0.335	0.099	-0.26
	**pC7**	**0.77**	**0.78**	-0.411	**0.755**	-0.267	-0.061	**-0.57**	0.13	-0.25
	**pC8**	0.13	0.32	**-0.505**	**0.631**	**-0.580**	**0.577**	0.131	0.03	-0.28
	**pC10**	0.41	**0.68**	-0.260	**0.575**	-0.164	0.103	-0.371	0.059	-0.08
Coast 5 m	**dPUA**	-0.041	0.056	0.287	-0.104	0.116	0.251	-0.425	-0.133	-0.0023
	**dC7**	0.26	-0.17	0.08	0.156	-0.060	0.218	-0.133	-0.085	0.188
	**dC8**	0.182	-0.204	-0.022	-0.128	-0.103	0.278	-0.041	-0.126	0.05
	**dC10**	-0.141	0.17	-0.12	0.073	0.346	0.083	0.105	-0.152	-0.34
	**pPUA**	**-0.955**	-0.219	**0.662**	-0.060	0.400	-0.322	**0.568**	**-0.94**	0.318
	**pC7**	**-0.917**	-0.173	**0.519**	-0.129	**0.569**	-0.480	0.463	**-0.89**	0.177
	**pC8**	**-0.917**	-0.226	**0.854**	0.016	0.102	-0.061	**0.655**	**-0.921**	0.484
	**pC10**	**-0.751**	-0.486	**0.821**	0.410	-0.406	0.476	**0.848**	**-0.791**	**0.827**
Gyre 5 m	**dPUA**	-0.148	0.125	0.0126	-0.05	0.132	0.044	0.113	0.359	-0.196
	**dC7**	-0.17	0.128	0.03	-0.04	0.141	0.076	0.101	0.454	-0.184
	**dC8**	-0.07	0.08	0.004	-0.06	0.081	-0.028	0.097	0.236	-0.173
	**dC10**	-0.278	0.217	-0.01	-0.05	0.223	0.168	0.160	0.283	-0.239
	**pPUA**	-0.012	0.01	**0.902**	0.227	-0.424	-0.455	-0.219	-0.109	-0.029
	**pC7**	**-0.852**	**-0.796**	0.287	-0.266	-0.497	**-0.730**	-0.158	0.75	0.816
	**pC8**	-0.194	-0.171	**0.897**	0.089	**-0.516**	**-0.529**	-0.202	0.046	0.14
	**pC10**	0.234	0.245	**0.892**	0.338	-0.310	-0.290	-0.199	-0.245	-0.267
Jet DCM	**pPUA tot**	**0.55**	**0.67**	-0.458	**0.748**	-0.317	0.189	-0.335	0.099	-0.26
	**pC7**	**0.77**	**0.78**	-0.411	**0.755**	-0.144	-0.061	**-0.570**	0.13	-0.25
	**pC8**	0.13	0.32	**-0.505**	**0.631**	**-0.580**	**0.577**	0.131	0.03	-0.28
	**pC10**	0.41	**0.68**	-0.26	**0.575**	-0.164	0.103	-0.371	0.059	-0.08
	**dPUA**	0.206	-0.181	0.034	0.18	-0.140	-0.058	-0.186	**0.598**	0.198
	**dC7**	0.259	-0.169	0.08	0.136	-0.195	-0.028	-0.162	0.333	0.227
	**dC8**	0.182	-0.204	-0.022	0.2	-0.137	-0.074	-0.206	**0.660**	0.193
	**dC10**	0.169	-0.119	0.072	0.133	-0.094	-0.039	-0.131	**0.522**	0.150
Coast DCM	**pPUA**	**-0.955**	-0.219	**0.662**	-0.06	0.400	-0.322	**0.568**	**-0.940**	0.318
	**pC7**	**-0.917**	-0.173	**0.519**	-0.129	**0.569**	-0.480	0.463	**-0.890**	0.177
	**pC8**	**-0.917**	-0.226	**0.854**	0.016	0.102	-0.061	**0.655**	**-0.921**	0.484
	**pC10**	**-0.751**	-0.486	**0.821**	0.41	-0.426	0.476	**0.848**	**-0.791**	**0.827**
	**dPUA tot**	-0.041	0.056	0.287	-0.104	0.116	0.251	-0.425	-0.133	-0.002
	**HD**	0.123	-0.131	0.128	0.156	-0.060	0.218	-0.133	-0.080	0.188
	**OD**	0.039	-0.003	**0.544**	-0.128	0.069	0.278	-0.041	-0.120	0.050
	**DD**	-0.36	0.362	-0.103	-0.339	0.346	0.083	0.105	-0.152	-0.340
Gyre DCM	**pPUA tot**	-0.012	0.01	**0.902**	0.227	-0.424	-0.455	-0.219	-0.109	-0.029
	**HD**	**-0.852**	**-0.796**	0.287	-0.266	-0.497	**-0.730**	-0.158	**0.750**	**0.816**
	**OD**	-0.194	-0.171	**0.897**	0.089	**-0.516**	**-0.529**	-0.202	0.046	0.140
	**DD**	0.234	0.245	**0.892**	0.338	-0.310	-0.290	-0.199	-0.245	-0.267
	**dPUA tot**	-0.148	0.125	0.0126	-0.05	0.132	0.044	0.113	0.359	-0.196
	**HD**	-0.17	0.128	0.03	-0.04	0.141	0.076	0.101	0.454	-0.184
	**OD**	-0.07	0.08	0.004	-0.06	0.081	-0.028	0.097	0.236	-0.173
	**DD**	-0.278	0.217	-0.01	-0.05	0.223	0.168	0.160	0.283	-0.239

Table S3: Sperman rank correlation coefficients performed on pPUA data and biological variables I (pigments) at the DCM of the different sites. High correlation are highlighted in grey. dPUA: dissolved PUA; pPUA: particulate PUA; dC7: 2*E*,4*E*/*Z*-heptadienal. dC8: 2*E*,4*E*/*Z*-octadienal; dC10: 2*E*,4*E*/*Z*-decadienal. Perid.: peridinin; Fx: Fucoxanthin; Viol.: violaxanthin; Prasin.; prasinoxanthin; Diadin.; diadinoxanthin; Allox.; alloxanthine; But-Fx:19′-butanoyloxyfucoxanthin. Hex-Fx: 19′-hexanoyloxyfucoxanthin. Significant values are highlighted in bold. "–" denotes undetected concentrations.

Station	pPUA	Chl*a*	Chlc_1	Chlc_2	Chlc_3	Perid.	But-Fx	Fx	Violax	Prasinox	Hex-Fx	Diadin.	Allox	Diatox.	Luteine
Jet	**pPUA**	0.91	-0.11	0.42	0.079	-0.256	0.003	0.918	0.441	0.977	0.964	0.901	0.842	-0.381	0.62
	**pC7**	0.946	-0.60	0.63	0.031	-0.023	-0.511	0.954	0.183	0.785	0.704	0.973	0.991	-0.414	0.502
	**pC8**	0.31	0.709	-0.07	0.217	-0.394	0.766	0.265	0.463	0.631	0.724	0.224	0.096	0.011	0.311
	**pC10**	0.51	-0.29	-0.21	-0.64	-0.432	-0.089	0.792	0.777	0.775	0.758	0.683	0.586	-0.896	0.991
Coast	**pPUA**	0.937	0.911	-0.982	0.783	0.03	0.587	0.851	0.65	0.533	0.984	0.803	0.99	0.908	
	**pC7**	0.988	0.809	-0.999	0.643	-0.171	0.413	0.728	0.485	0.352	0.929	0.668	0.986	0.806	
	**pC8**	0.769	0.996	-0.865	0.945	0.361	0.823	0.977	0.866	0.784	0.986	0.956	0.929	0.995	
	**pC10**	0.475	0.956	-0.616	0.998	0.683	0.975	0.986	0.99	0.959	0.854	0.996	0.725	0.958	
Gyre	**pPUA**	-0.214		-		-0.374	-0.456		-		-0.227	-0.351	-	-0.141	-
	**pC7**	0.494		-		-0.193	0.178		-		0.188	0.481	-	0.31	-
	**pC8**	-0.047		-		-0.33	-0.34		-		-0.107	-0.191	-	-0.004	-
	**pC10**	-0.385		-		-0.36	-0.554		-		-0.314	-0.527	-	-0.256	-

Table S4: Spearman rank correlation coefficients performed on pPUA data and biological variables II (biovolume of taxonomical categories of large size phytoplankton) at the DCM of the different sites. High correlation is highlighted in grey. Significance level *p< 0.05; **p<0.01. dPUA: pPUA: particulate PUA; dC7: 2*E*,4*E*/*Z*-heptadienal. dC8: 2*E*,4*E*/*Z*-octadienal; dC10: 2*E*,4*E*/*Z*-decadienal. Significant values are highlighted with grey area.

		**Diatoms**	**Cocolitophorids**	**Silicoflagellates**	**Dinoflagellates**	**Others**
**Jet**	**pPUA**	0.730	-0.425	0.881*	0.043	0.466
	**pC7**	0.831*	-0.326	0.897*	0.057	0.524
	**pC8**	0.463	-0.536	0.622	0.117	0.338
	**pC10**	0.568	-0.292	0.945**	-0.215	0.282
**Coast**	**pPUA**	-0.086	-0.157	0.244	-0.041	-0.116
	**pC7**	-0.174	-0.208	0.339	-0.189	-0.203
	**pC8**	0.021	-0.106	0.026	0.171	-0.009
	**pC10**	0.446	0.286	-0.029	0.645*	0.423
**Gyre**	**pPUA**	-0.559	-0.43	0.092	-0.483	-0.514
	**pC7**	-0.193	-0.429	0.106	-0.687	-0.544
	**pC8**	-0.591	-0.524	0.109	-0.611	-0.62
	**pC10**	-0.542	-0.33	0.068	-0.318	-0.392
**Total**	**pPUA**	0.338	-0.079	0.569*	-0.103	0.273
	**pC7**	0.503*	0.085	0.744*	-0.073	0.414
	**pC8**	0.333	-0.103	0.566*	-0.058	0.327
	**pC10**	0.578*	0.0109	0.746*	-0.065	0.509
